# Supplementary material for: Memristor-based feature learning for pattern classification
Source: Nat Commun. 2025 Jan 21;16:913. doi: 10.1038/s41467-025-56286-y (PMC11751336; doi:10.1038/s41467-025-56286-y)
Supplement: Supplementary file 1 — Supplementary Information [file 41467_2025_56286_MOESM1_ESM.pdf]

Supplementary Materials for

Memristor-based feature learning for pattern classification

Tuo Shi[1], Lili Gao[1], Yang Tian[1], Shuangzhu Tang[1], Jinchang Liu[1], Yiqi Li[1], Ruixi Zhou[1], Shiyu Cui[1], Hui Zhang[1], Yu Li[2], Zuheng Wu[3], Xumeng Zhang[2], Taihao Li[1], Xiaobing Yan[4*] and Qi Liu[2*]

[1] Zhejiang Laboratory, Hangzhou 311122, China.
[2] Frontier Institute of Chip and System, Fudan University, Shanghai 200433, China.
[3] School of Integrated Circuits, Anhui University, Hefei 230601, China.
[4] Key Laboratory of Brain-like Neuromorphic Devices and Systems of Hebei Province, Hebei University, Baoding 071002, China.

[*]Corresponding author. E-mail: yanxiaobing@ime.ac.cn; qi_liu@fudan.edu.cn

Contents

I: Supplementary Notes

1. DDK operation evaluation
2. Benchmark of the MFCC hardware
3. Software toolchain and hardware system
4. Implementation of neural networks
5. Calculation of the number of parameters and operations in neural network
6. The effect of endurance on neural network performance
7. Hardware benchmark

II: Supplementary Figures and Captions

Fig. S1 | Typical RS curves obtained by the simplified drift-diffusion model

Fig. S2 | Influences of α and β on the output of the proposed model

Fig. S3 | Structure and composition characterization of the TiN/TaO_x/HfO_x/TiN memristor device with HRTEM

Fig. S4 | Finite-element modeling of the TiN/TaO_x/HfO_x/TiN device

Fig. S5 | The temperature distribution for the TiN/TaO_x/HfO_x/TiN device during the reset (a-d) and set processes (e-h).

Fig. S6 | The oxygen vacancy distribution for the TiN/TaO_x/HfO_x/TiN device during the reset (a-d) and set processes (e-h).

Fig. S7 | The pulsed *I-V-t* curves of the memristor

Fig. S8 | Device-to-device test for tunability of parameters α and β

Fig. S9 | Comparison of cepstrum method and the proposed method in 1-D raw data processing

Fig. S10 | Architecture of a hardware-software system for deployment

Fig. S11 | Memristor-based hardware system

Fig. S12 | Device programming and MVM output

Fig. S13 | DDK neural network architecture

Fig. S14 | Feature maps experimentally obtained with memristors

Fig. S15 | *In-situ* training flowchart

Fig. S16 | Influence of cycle-to-cycle variation on DDK network in audio classification

Fig. S17 | Quantization performances of the DDK network on SITW dataset

Fig. S18 |Endurance test of the memristor device

Fig. S19 |The relation of latency and accuracy in the endurance test in audio classification task

Fig. S20 | Comparison of compiler-generated mapping strategies

III: Supplementary Tables

Table S1 | Material parameters in finite-element simulation

Table S2 | Comparison of various hardware for MFCC feature extraction

Table S3 | Training hyperparameters of DDK neural networks

Table S4 | Training hyperparameters and physical parameters of memristor array

Table S5 | Training hyperparameters of convolutional and MFCC-based neural networks

Table S6 | Comparison of DDK network with other DNNs in terms of operation numbers (MACs) in feature extraction and classification

Table S7 | The number of parameters and operations of MFCC

Table S8 | Comparison of various layer types based on computational efficiency metrics (per-layer complexity, minimum number of sequential operations and maximum path lengths)

Table S9 | Hardware parameters at 180 nm technology node in simulation

Table S10 | Detailed metrics of each circuit module of the memristive hardware system in the mapping strategies

Table S11 | Benchmark of the mapping strategies

Table S12 | The number of operations of the DDK network for SITW

Table S13 | The number of operations of the CNN for SITW

Table S14 | The number of operations of the DDK network for UCF

Table S15 | The number of operations of the CNN for UCF

IV: Supplementary References

I: Supplementary Notes

1. DDK operation evaluation

The energy of a single DDK operation of the TiN/TaO_x/HfO_x/TiN cell is calculated as follows:

$$Energy_{DDK} = \frac{V_{RESET}^2}{R} t_{RESET}, \tag{S1}$$

where V_{RESET} and t_{RESET} are the amplitude and width of the RESET voltage pulse, respectively. R is the resistance of the device. In our experiment, the amplitude of the RESET pulse is typically between 1.5 and 2.5 V with a pulse width of 10 ns, the resistance range of a typical TiN/TaO_x/HfO_x/TiN cell is from 1 to 100 KΩ. Therefore, we choose V_{RESET} = 2 V, t_{RESET} = 10 ns and R = 50 KΩ in our evaluation, resulting in an energy consumption of 0.8 pJ.

2. Benchmark of the MFCC hardware

MFCC and the proposed DDK method both extract features by the grain size of frame. When comparing performance among different hardware, we focus on the energy consumption per frame. From the point of fairness, the hardware performance benchmark only takes into account the feature extraction, other stages like classification will be ignored. And for the different kinds of hardware in the reference papers, like ASIC, FPGA, DSP, GPU, and CPU, the energy consumption is calculated in different ways. Here we represent the computing details and modify some parts of the benchmark in Table S2.

(1) ASIC: These ASIC circuits are newly proposed, and the performance details are given in the paper. The energy consumption can be calculated indirectly or obtained directly based on the information provided in the papers.

For ASIC[1], the latency of MFCC per frame is 16 ms (4-stage pipeline with 64 ms total latency) and

the power of MFCC is 0.34 μW. As a result, the energy consumption of MFCC per frame is 5.44 nJ (16 ms×0.34 μW).

For ASIC[2], the latency of MFCC per frame is calculated from the data in the paper, which is 2 ms (calculated from the time sampled from Figure in the reference paper, the start time of MFCC is 4.5 ms, and the end time of MFCC is 6.5 ms). In the same way, the average power of MFCC is calculated, which is 21.23 μW (11 points of the power consumption sampled from 4.5 ms to 6.5 ms to calculate the average power of MFCC, which are 21 μW, 22.5 μW, 26 μW, 29 μW, 30 μW, 30 μW, 29 μW, 15 μW, 13 μW, 9 μW, 9 μW). As a result, the energy consumption of MFCC per frame is 42.46 nJ (2 ms×21.23 μW).

For ASIC[3], the performance can be directly obtained. The energy consumption of MFCC per frame is 0.72 μJ, and the latency of MFCC per frame is 45.79 μs.

(2) FPGA: FPGAs are mature products, and their performance details may not be given in the paper. However, we can evaluate power using AMD Power Estimator (XPE) to get a rough power consumption result, which is based on the resources used in the paper. Then the energy consumption of the MFCC per frame can be calculated using the power and the latency mentioned in the paper.

For FPGA[4], the MFCC latency is 14601 cycles per frame, which is 14601×20 ns (clock frequency is 50 MHz) = 292 μs. In AMD Power Estimator (XPE), we set the device as XC4VLX15 (the same device as the one used in the paper), and the logic part as resources mentioned in the paper, such as the number of LUT and Flip flops, and then we get an estimated power (172 mW). Finally, the energy

consumption of MFCC per frame is calculated to be 50.22 μJ (172 mW×292 μs).

For FPGA[5], the MFCC latency is directly mentioned in the paper, which is 285.44 μs. In AMD Power Estimator (XPE), we set the device as XC3S2000, and the logic part as resources mentioned in the paper, such as the number of LUT and Flip flops, and then we get an estimated power (234 mW). Finally, the energy consumption of MFCC per frame is calculated to be 66.79 μJ (234 mW×285.44 μs).

(3) DSP, GPU, and CPU: DSPs, GPUs, and CPUs are also mature products, and their performance details may not be given in the paper. However, their power consumptions can be evaluated from the product specifications. Then, the energy consumption of MFCC can be simply calculated with the latency of MFCC mentioned in the paper.

For GPU[6], the MFCC latency per frame is 9.17 μs. The power consumption of GTX580 GPU from product specification is 244 W[7]. Finally, the energy consumption of MFCC per frame is 2.24 mJ (244 mW×9.17 μs).

For DSP[8], the MFCC latency is 0.912 ms. The power consumption of TMS320C6713 from product specification is 1.067 W[9]. Finally, the energy consumption of MFCC per frame is 0.973 mJ (1.067 W×0.912 ms).

For CPU[5], the MFCC latency is 238.77 μs. The power consumption of Intel P4 CPU from product specification is 57.8 W[10]. Finally, the energy consumption of MFCC per frame is 13.8 mJ (57.8

mW×238.77 μs).

3. Software toolchain and hardware system

The hardware system for chip test and demonstration is shown in Fig. S11. The system is designed and developed in cooperation with the College of Electronic Science and Technology, National University of Defense Technology (Changsha, China). It consists of a socket for chip placement, a field programmable gate array (FPGA) chip for configuration, multiple switches for signal selection, analog-to-digital converters (ADCs) for electrical response measurement, digital-to-analog converters (DACs) for voltage programming, and ethernet port for communication between an upper computer and FPGA. In the implementation of the neural network on hardware, the activation functions and pooling operations are realized in the upper computer, while the DDK and MVM operations are implemented in the memristor crossbar array.

Although manual deployment of neural network models on hardware is feasible, it is time-consuming, inflexible, and not user-friendly. Here we developed a software toolchain for automatic deployment of neural network models on memristive hardware. The toolchain consists mainly of an imprecision-based neural network training framework, a compiler, and a software-hardware interface.

The framework is developed for neural network model construction and optimization based on the imprecise nature of analog computing, e.g., memristor-based computing. Here we considered three main imprecision factors, i.e., available conductance range, output variation and device failure. Because of the limited conductance range of memristor, the neural network is trained with clipped weight. The range of clipped weight is from –1 to 1, corresponding to a device conductance range from 40 to 200 μS. If the weight is larger than 1 or smaller than –1, it is forced to be 1 or –1, respectively. The variation of MVM output shown in Fig. S12 is critical, especially when the network is deep, since the accumulation of MVM output errors layer-by-layer deteriorates network performance rapidly. According to experimental results, the MVM output variation follows roughly

normal distribution. We use mean and standard deviation from the experimental data in Fig. S12 to train the network. Device failure is an unusual but critical issue in memristive crossbar arrays. The criterion for a failed device in our work is that the device conductance cannot be programmed to >180 μS or <50 μS, even with a very large SET or RESET pulse, respectively. When such device failure is detected, its conductance is programmed to <120 μS to minimized its influence on MVM output. In network training, the weight represented by this failed device is frozen. In our experiment, a device failure rate of 10% is assumed.

The compiler is used to automatically deploy optimized neural network model from the framework to memristive hardware. Its architecture follows the pipe-and-filter pattern[11]. The front-end of the compiler deals with graph-level optimization, including operator fusion, static memory planning and layout transformation. The back-end performs tensor-level optimization, including partition, scheduling, and intrinsic mapping. Hardware primitives are encapsulated into low-level driver application programming interfaces (APIs) to be called by high-level applications. An operator library is also built based on these driver APIs. This library contains specifically optimized implementations of critical operators, e.g., DDK, MVM.

4. Implementation of neural networks

Audio recognition

Speakers in the Wild (SITW) database

The SITW dataset was specifically designed for the purpose of text-independent speaker recognition. The dataset consists of a comprehensive collection of 299 audio recordings from various speakers, sourced from open-source media. Each speaker is represented by an average of 8 sessions. Here a total of 10 recordings from 10 speakers in the evaluation part were selected in the SITW, with each recording lasting for 10 seconds. 80% of the audio frames were used for training and the remaining 20% for testing.

Network architecture

State-of-the-art sound recognition systems mostly rely on cepstral-based feature extraction methods that collapse high dimensional sound waves into low dimensional feature vectors. MFCC is the most successful and widely used feature since it usually focuses on the most informative part of signal, is low on complexity and well-tested. After MFCC feature extraction, a classifier is used for pattern classification. Recently, there is a trend to use CNN in the field of audio recognition. There are two kinds of network architectures for audio recognition: sample-level and frame-level CNN. The sample-level CNN is an end-to-end learning method to learn hierarchical representations from raw data. In this work, we used FC layers after MFCC feature for pattern classification. We also designed a sample-level CNN for WMMS and SITW datasets according to the network structure proposed in literature[12]. The structures of the MFCC-based network and CNN are detailed in Table S5.

Text classification

AG News

The AG News dataset was collected by an academic news search engine. There are as many as 2000 news data sources. The AG News dataset contains four types of news: world, sports, business and technology. The dataset consists of 120000 training sets and 7600 test sets. Here, 10000 samples are selected randomly from 120000 training sets as validation sets.

Network Architecture

Text classification is a fundamental task in the field of natural language processing (NLP). When dealing with sequences, recurrent neural networks are typically used, especially some of its variants such as LSTM, GRU and so all. Among various models, the RCNN proposed by Siwei Lai et al. is in most cases a benchmark[13]. Recently, transformer and its variants are widely used for NLP[14]. In this paper, we adopt the LSTM-based RCNN and transformer for AGNews text classification.

Image recognition

Modified National Institute of Standards and Technology (MNIST)

The dataset used in this study was developed by the National Institute of Standards and Technology (NIST) and consists of 70,000 grayscale images, with 60,000 images used for training and 10,000 for testing. Each image is a 28×28-pixel representation of a handwritten number. The training set is composed of handwritten numbers from 250 individuals, with an equal proportion of high school students and Census Bureau staff. The test set is also composed of handwritten numbers from the same two groups, but care has been taken to ensure that individuals in the test set are not included in the training set. This dataset is widely used in the literature to evaluate the performance of machine learning algorithms for image classification tasks.

Network architecture

Currently, 2-D convolutional neural networks are typically used for image classification. LeNet-5, a model specifically applied to handwriting recognition, is one of the representative convolutional neural networks[15]. In this paper, LeNet-5 is used for MNIST dataset classification.

Video recognition

UCF

The UCF dataset is a widely used benchmark in the field of video recognition due to its close resemblance to actual daily life scenarios and inclusion of a range of basic and complex human movements, interactions, instruments performance and sports activities. The dataset contains more than 12,000 videos, comprising of 101 distinct action behaviors. The UCF dataset is further categorized into five distinct action categories based on the nature of the action performed: basic human movements, human-to-human interaction, human-object interaction, musical instrument performance and sports. Additionally, each video is labelled and organized into 25 groups, with each group comprising of 4-7 videos depicting the same action. In this work, a total of 10 sport categories

in UCF, each containing about 100 action videos for classification. 80% and 20% of the videos are used for training and testing, respectively.

Network architecture

Currently, 2-D or 3-D convolution kernels are typically used for action recognition. For 2-D CNN, temporal information is propagated across frames through temporally recurrent layers or feature aggregation, but these methods are limited in efficiency. On contrary, 3-D CNN directly creates hierarchical representations of spatiotemporal data. However, the problem with these models is that they have more parameters than 2-D models, which makes training more difficult. In this paper, C3D network[16], a 3-D convolution kernel-based network widely used in video stream classification, is used for UCF-10 dataset classification.

3-D object recognition

ModelNet

The ModelNet dataset comprises 127,915 3-D CAD images belonging to 662 categories. ModelNet40 and ModelNet10 are subsets used for 3-D object recognition benchmarks. ModelNet40 includes 4,899 images categorized into 40 classes, while ModelNet10 has 4,899 images classified into 10 groups.

In this paper, 100 randomly selected 3-D CAD images from each of the 10 categories in ModelNet10 are used for classification (80% for training and 20% for testing).

Network architecture

3-D CNN is one of the main techniques for 3-D object recognition. Wu et.al. proposed 3D ShapeNet[17] based on convolutional deep belief network, which is a benchmark for 3-D CNN-based models. Maturana and Scherer proposed VoxNet[18], which is an improved version of 3D ShapeNet. We adopted the network architecture of VoxNet. VoxNet converts point cloud into binary voxels as input

to the network. The size of binary pixels is 32×32×32.

DDK Neural Network

The DDK neural network proposed in this paper consists of DDK layer for feature learning and FC layer for classification, as shown in Fig. S13. The number of FC layers varies for different applications. For the application of audio recognition, the steps of processing the raw audio waveform are as follows: (1) an audio waveform is segmented into m waveform segments. Each segment is a n-dimensional vector. Then a n × m 2-dimensional waveform is formed; (2) The n × m matrixes of all audio waveforms are stacked together along one dimension, e.g., the dimension of n, and form the input of the network. For the SITW and MNIST dataset, the network has only 1 FC layer. For the AGNews dataset, the network has only one FC layer followed by 1-D max-pooling layer. For the UCF and ModelNet, the network structures have 3 FC layers. For the UCF dataset, the DDK is performed on each video frame (80 × 60, width × height) along the width dimension. In contrast to the network for the SITW, AG News and MNIST dataset, where a unified α and β is used for feature learning, each feature learning dimension in the network for UCF has its own α and β to improve performance. As a result, the number of DDK layer parameters for UCF is 60 × 2 = 120. For the AG News dataset, the word vectors (64 × 300, word length × word dimension) for each text are input to the DDK layer. The DDK is carried out in the word dimension and word vector dimension. For the ModelNet dataset, the data of X and Z axes in the binary voxels are flattened to form 2-D voxels. DDK is carried out on the Z axis. The number of DDK layer parameters is 64.

5. Calculation of the number of parameters and operations in neural network

The number of parameters that need to be trained in a neural network model is used to measure the size of the model. For convolutional layer, the number of parameters is calculated as follows:

$$\text{Params}_{\text{Conv}} = C_{out} \times (h \times w \times C_{in} + 1) \quad \text{(S2)}$$

Where h and w are height and width of convolutional kernel, C_{in} is the number of channels of

input feature map, and C_{out} is the number of output feature map.

For fully connected layer, the number of parameters is calculated as follows:

$$\mathrm{Params_{FC}} = D_{out} \times (D_{in} + 1) \quad \text{(S3)}$$

Where, D_{in} is input dimensionality and D_{out} is output dimensionality.

For feature learning layers based on memristors, alpha and beta are used as parameters for this layer.

MACs (multiply–accumulate operations) refers to the number of multiplication and addition operations in a neural network, which is used to measure the computational complexity of a model. 1 MAC consists of a multiplication operation and an addition operation. For the convolutional layer, MACs are calculated as follows:

$$\mathrm{MAC_{Conv}} = \mathrm{H} \times \mathrm{W} \times C_{out} \times (h \times w \times C_{in} + 1) \quad \text{(S4)}$$

Where H and W are height and width of output feature map.

For fully connected layer, MACs are calculated as follows:

$$\mathrm{MAC_{FC}} = D_{out} \times D_{in} \quad \text{(S5)}$$

For the DDK layer, MACs are calculated as follows:

$$\mathrm{MAC_{DDK}} = 2D_{in} \quad \text{(S6)}$$

For the MFCC component in neural network, its parameters and operations at each processing stage is listed in Table S7. Here the FFT size is 512, the number of MFCC feature is 40 and the number of Mel filters is 40. The total number of parameters of MFCC is 10793. The total number of additions and multiplications in MFCC are 30784 and 12864, respectively. Thus, the operation number equals to 12864 MACs plus 17920 additions. For simplicity, we only count the 12864 MACs in the comparison in Fig. 5b.

6. The effect of endurance on neural network performance

The endurance of RRAMs may be an issue causing performance degradation in their application scenarios, such as memory and artificial neural network. Therefore, the trade-offs between latency, accuracy, and endurance are further investigation. Since the latency involved in re-writing the RRAMs is directly determined by the pulse width that is used by the writing pulse to program the device, the trade-offs are studied by investigating the relations between pulse width, endurance and neural network accuracy. To be specific, we study the influence of pulse width on device endurance, and then the impact of conductance degradation found in the endurance test on the classification accuracy of the DDK neural network. Since the re-writing occurs primarily in the DDK layer, the device conductance in the classification layers is assumed to be constant to simplify the study and highlight the effect of re-writing. The study is performed based on simulation using models extracted from experimental data.

We firstly study the influence of pulse width on device endurance. To make the endurance test more reliable, the endurance of the device is tested using the same method as in the DDK feature extraction. In this method, a large and constant gate voltage is applied so that the primary role of gate voltage is to select the specific device but not control the conductance states by limiting the compliant currents. Therefore, the conductance states are determined by the voltages applied on BL and SL. For the LTP process, the voltage of WL for set operation is fixed at 2 V, while the voltage of BL increases gradually (from 0.7 V to 1.7 V) and SL is ground. For the LTD process, the voltage of WL for reset operation is fixed at 3 V, while the voltage of SL increases gradually (from –1.4 V to –2.4 V) and BL is ground. The pulse width in the writing operation is fixed at 50 ns, 200 ns, 500 ns, respectively. The read operation uses a fixed voltage (0.2 V) and pulse width (20 μs). Fig. S18 shows the endurance test results of the devices. Fig. S18a shows the LTD and LTP characteristics around initial, 10^3, 10^6, 10^7,

and 10^9 pulses. The conductance range and on/off ratio of at least 5 devices for each pulse width configuration in the endurance test are statistically calculated in Fig. S18b and Fig. S18c, respectively. Though the dynamic range and on/off ratio degrade as the increase of update pulses, there is an on/off ratio larger than 1 after 10^9 update pulses. Previous research has demonstrated that, in contrast to full-window switching under strong programming pulses, employing weaker weight update pulses in the low-resistance region can enhance the incremental switching cycles of RRAM by over five orders of magnitude, reaching approximately 10^{11} cycles[19]. Although the switching region in our endurance test is not constrained in a low resistance region, the conclusions are still in consistence with the previous study. Besides, it can also be observed that the high resistance states in the three pulse configurations all shows firstly an upward then a downward trend, and the trend of the 500 ns configuration is flatter in the pulse number $\leq 10^7$ than the other two. This may be attribute to the effect of the "second order memristor", where non-gradual resistive switching behavior is typically observed under long pulse width, because even a single set pulse can result in substantial heat accumulation if its duration is sufficiently extended[20].

Secondly, to study the impact of conductance degradation found in the endurance test on the classification accuracy of the DDK neural network, the effect of conductance degradation on neural network parameters, namely, α and β, should be modeled. Based on experimental data in Fig. 2c and 2d, we respectively demonstrate the specific impacts of different pulse widths and different initial resistances on the α and β parameters. By observing Fig. 2c, it can be clearly seen that as the pulse width gradually increases, the β exhibits an increasing trend, while the α remains almost unchanged throughout this process. Similarly, in Fig. 2d, as the initial resistance continues to rise, the β also shows a corresponding increasing trend and eventually stabilizes, while the α value remains relatively

stable.

Given this, we will next conduct an in-depth analysis of the specific impacts of pulse width and initial resistance on the β, and explore how these impacts further affect classification accuracy. During the network inference process, we can adjust parameters such as pulse amplitude, initial resistance, and pulse width to match the β of the device with the β obtained from network training. However, it is worth noting that after multiple operations, the β will inevitably undergo degradation. Therefore, we analyze in detail the effect of β decay on the accuracy with the increase of pulse number at different pulse widths.

(1) Evaluation of the changes in initial resistance (high conductance state) at different pulse widths. Here we use the average high conductance states of the at least 5 test devices for each pulse configuration as the initial resistance. Changes in initial resistance at different pulse widths are shown in Fig. S19a. The initial resistance shown on the y-axis in Fig. S19a is based on the average of the initial resistances measured from average at least 5 devices in Fig. S18. In consistence with previous analysis, the fluctuation of the initial resistance for pulse number $\leq 10^7$ is the smallest when the pulse width is 500 ns. After 10^7 cycles, the initial resistances of the three configurations all degrades.

(2) Modeling the relation between initial resistance and β at arbitrary pulse width. For simplicity, we construct a linear model between initial resistance and β using the relation of initial resistance and β at 5 KΩ, 10 KΩ and 15 KΩ initial resistances in Fig. 2d. β exhibits an increasing trend as the initial resistance increases.

Since this extracted linear model is based on the data measured at pulse width of 100 ns, this model

should be extended to situations where arbitrary pulse width is used. The linear model we construct at pulse width of 100 ns is shown as follows:

$$\beta = (9.66 \times 10^{-5}) \times R_{init} - 0.21 \tag{S7}$$

where R_{init} is the initial resistance. We assume that at arbitrary pulse width, the slope of the extended linear model is unchanged, but the data point it passes through is determined by another linear model between pulse width and β, using the experimental data in Fig. 2c. The linear model between pulse width and β is shown as follows:

$$\beta = 0.00134 \times T + 0.14378 \tag{S8}$$

where T is the pulse width. Using the slope and the data points it passes through, the extended linear model that describes the relation between initial resistance and β at arbitrary pulse width can be constructed. The extended linear model at pulse width of 50 ns, 200 ns and 500 ns is shown as S9-S11:

$$\beta = (9.66 \times 10^{-5}) \times R_{init} - 0.27 \tag{S9}$$

$$\beta = (9.66 \times 10^{-5}) \times R_{init} - 0.07 \tag{S10}$$

$$\beta = (9.66 \times 10^{-5}) \times R_{init} + 0.33 \tag{S11}$$

From the experimental data, the β values typically fall within the range of 0 to 1, for β values that exceed this range, they are truncated directly.

(3) Modeling the change rate of β at arbitrary pulse widths. The change rate of β with the number of pulses at different pulse widths can be derived from the extended linear model between β and the initial resistance, as well as the degradation process of initial resistance with pulse number (Fig. S19a). As shown in Fig. S19b, the rate of β change fluctuates the most at pulse width of 50 ns and the least at pulse width of 500 ns. Because, even though the initial resistance experiences a relatively high rate

of change after the number of pulses reaches 10^9 at pulse width of 500 ns, the β value is truncated, resulting in a small change rate.

(4) Modeling the decay process of β obtained from the pre-trained network. Here, we set the decay rate of the β obtained from the pre-trained network to be the change rate of β in step (3). Fig. S19c shows the decay process of the β obtained from the pre-trained network with an increasing number of pulses at different pulse widths. It can be observed that under different pulse widths, the decay of β exhibits a trend of first increasing and then decreasing. Specifically, when the pulse width is 50 ns, the fluctuation amplitude is the largest, while when the pulse width reaches 500 ns, the fluctuation amplitude is the smallest.

(5) Study of β decay on classification accuracy. We test the impact of β decay on classification accuracy on the SITW dataset in a 10-speaker recognition task at different pulse widths (as shown in Fig. S19d). It can be observed that the classification accuracy exhibits a downward trend as the number of pulses increases for all pulse widths. Particularly, when the pulse width is 50 ns, the decline in classification accuracy is the most significant. As the pulse width increases, the downward trend in classification accuracy gradually slows down. This indicates that in endurance test, the pulse width used for re-writing has a significant impact on accuracy degradation. Specifically, small pulse width has low latency, but the degradation in classification accuracy is significant and its endurance is poor; large pulse width has high latency, but the degradation in classification accuracy is less pronounced and its endurance is good, especially when the pulse number is $\leq 10^7$.

7. Hardware benchmark

Using the experimental data, a simulator evaluates the memristor-based hardware system

performances in accordance with mapping strategies offered by the compiler. The simulator compares how well different mapping strategies work with a single task when given the DDK neural network model (SITW) and the necessary hardware information (Table S9) at a commercial 180 nm technology node.

Fig. S20 illustrates four mapping strategies on crossbar arrays. Input data is entered into the array through the rows, while analog output results can be read through the columns. Strategy A utilizes three arrays with 64 rows and 64 columns. Additionally, three single arrays share the 4 ADCs, 4 DACs, and 2 transimpedance amplifiers (TIAs). Strategy B makes use of a single array with 128 rows and 256 columns, requiring 8 ADCs, 8 DACs, and 4 TIAs. Strategy C and strategy D are both applied to multiple arrays. Each array has the same number of ADCs, DACs, and TIAs for highly efficient parallelization. Strategy C employs 6 arrays, and each array is 64×64 with 4 ADCs, 4 DACs, and 2 TIAs. Strategy D, on the other hand, employs 4 arrays, and each array is 128×128 with 8 ADCs, 8 DACs, and 4 TIAs.

Table S10 lists the detailed metrics, including area, energy, and latency, of the four strategies. The DDK layer is a diagonal matrix. The DDK layer is converted into a 128×128 diagonal matrix in Strategies B and D, which both support 128 inputs in one cycle. The DDK layer is optimized by two 64×64 diagonal matrixes in Strategies A and C because the circuit modules of Strategies A and C support 64 inputs in one cycle. Therefore, compared with Strategies B and D, Strategies A and C save 50% of arrays in the DDK layer. As a result, the area and the energy consumption of the array in Strategies A and C are less than those in Strategies B and D.

The circuit modules of Strategies A and C support 64 inputs in one cycle, resulting in the same utilization of the items (ADCs, DACs, and TIAs) in the simulation. Consequently, the simulation results reveal that both Strategies A and C consume the same amount of energy. Similarly, Strategy B consumes the same amount of energy as Strategy D in the simulation, supporting 128 inputs in one cycle. Strategies C and D aim to maximize parallel computing capabilities for reducing the latency of task processing, making them require more ADCs, DACs, and TIAs than Strategies A and B. In general, among the four strategies, Strategy A is the most area-saving strategy (3.23 mm^2), it occupies seven times less area than Strategy D (22.68 mm^2).

The DDK neural network model includes one DDK layer and two dense layers. The DDK layer includes 128×128×4 = 65536 operators, and two dense layers include (129×16+17×10)×2 = 4468 operators. Therefore, the total number of operations in a single inference task is 70004 ops. The performance, power, area, energy efficiency, and performance density of the four mapping strategies are shown in Table S11. After comparing these four strategies, it is indicated that strategy A is the most area-saving strategy, strategies B and D achieve high energy-efficiency (2.77 TOP $s^{-1}W^{-1}$), and strategy C has a large performance density (210.28 GOP s^{-1} mm^{-2}).

The evaluation process of the memristor neural network hardware is as follows:

For each device, the read voltage pulse is 0.2 V, 20 ns. The average device conductance is 50 μS. The energy consumption of a single array cell is:

$$E_{cell} = 20\ ns \times (0.2\ V)^2 \times 50\ \mu S = 40\ fJ \qquad \text{(S12)}$$

For DDK layer, the TiN/TaO_x/HfO_x/TiN cell are estimated to be 10 ns, using an average voltage of 2 V and an average conductance of 20 μS. The energy consumption of a DDK operation in DDK layer

is:

$$E_{DDK\_operation} = 10\,ns \times (2\,V)^2 \times 20\,\mu S = 0.8\,pJ \quad \text{(S13)}$$

Considering adding the external circuit part. The maximum energy cost of a single DAC, ADC, and TIA in Table S9, could be estimated as:

$$E_{DAC} = 12.6\,mW \times 5\,ns = 63\,pJ \text{ (180 nm, 8-bit, 200 MHz)} \quad \text{(S14)}$$

$$E_{TIA} = 7.2\,mW \times 0.4\,ns = 2.88\,pJ \text{ (180 nm, 2.5 GHz)} \quad \text{(S15)}$$

$$E_{ADC} = 8.5\,mW \times 20\,ns = 170\,pJ \text{ (180 nm, 8-bit, 200 MHz)} \quad \text{(S16)}$$

As shown in Table S12, for the DDK network for SITW dataset, the number of input neurons is 128, so the number of DDK operation ($n_{DDK\_operation}$) used and the number of DACs (n_{DAC}) used is 128. The total of the number of output neurons in each layer is 154, thus the number of TIAs used is $n_{TIA} = 154$. The 10 output neurons at the output layer could have their TIA signals converted to digits with $n_{ADC} = \text{ceil}\left(\frac{n_{output\ dim}}{20\,ns \times 0.2\,GHz}\right) = 3$. We set the number of devices used by the DDK layer is 1, and the number of weights in the classification layer for the DDK network is 2234, so the total number of devices required is the sum of the number of devices in the DDK layer and the number of devices in the classification layer, that is, $n_{total\_cell} = n_{cell} + n_{DDK\_cell} = 2234 \times 2 + 1$. Therefore, the maximal total energy consumption for the DDK network for SITW dataset could be estimated as:

$$E_{total} = n_{DDK\_operation} \times E_{DDK\_operation} + n_{cell} \times E_{cell} + n_{DAC} \times E_{DAC} + n_{TIA} \times E_{TIA} + n_{ADC} \times E_{ADC} = 128 \times 0.8\,pJ + 2234 \times 2 \times 0.04\,pJ + 128 \times 63\,pJ + 154 \times 2.88\,pJ + 3 \times 170\,pJ = 9206.48\,pJ \quad \text{(S17)}$$

The area of a 1T1R single array is 25×25 μm^2. The area of a single DAC, ADC, and TIA is 0.068 mm^2, 0.05 mm^2 and 0.34 mm^2, respectively. The area used for the DDK network for SITW dataset could be estimated as:

$$Area_{total} = n_{DAC} \times DAC_{area} + n_{ADC} \times ADC_{area} + n_{TIA} \times TIA_{area} + (n_{DDK\_cell} + n_{cell}) \times$$

$$Cell_{area} = 128 \times 0.068\, mm^2 + 3 \times 0.05\, mm^2 + 154 \times 0.33\, mm^2 + (1 + 2234 \times 2) \times 6.25 \times 10^{-4}\, mm^2 = 62.467125\, mm^2 \quad \text{(S18)}$$

As shown in Table S13, S14 and S15，the maximal total energy consumption for the SL-CNN network for SITW dataset could be estimated as:

$$E_{total} = n_{cell} \times E_{cell} + n_{DAC} \times E_{DAC} + n_{TIA} \times E_{TIA} + n_{ADC} \times E_{ADC} = 662656 \times 2 \times 0.04\, pJ + 1600 \times 63\, pJ + 210698 \times 2.88\, pJ + 3 \times 170\, pJ = 761132.72\, pJ \quad \text{(S19)}$$

The area used could be estimated as:

$$Area_{total} = n_{DAC} \times DAC_{area} + n_{ADC} \times ADC_{area} + n_{TIA} \times TIA_{area} + n_{cell} \times Cell_{area} = 1600 \times 0.068\, mm^2 + 3 \times 0.05\, mm^2 + 210698 \times 0.33\, mm^2 + 662656 \times 2 \times 6.25 \times 10^{-4}\, mm^2 = 70467.61\, mm^2 \quad \text{(S20)}$$

The maximal total energy consumption for the DDK network for UCF dataset could be estimated as:

$$E_{total} = n_{DDK\_operation} \times E_{DDK\_operation} + n_{cell} \times E_{cell} + n_{DAC} \times E_{DAC} + n_{TIA} \times E_{TIA} + n_{ADC} \times E_{ADC} = 4800 \times 0.8pJ + 649600 \times 2 \times 0.04pJ + 4800 \times 63pJ + 5578 \times 2.88pJ + 3 \times 170pJ = 374782.64\, pJ \quad \text{(S21)}$$

The area used could be estimated as:

$$Area_{total} = n_{DAC} \times DAC_{area} + n_{ADC} \times ADC_{area} + n_{TIA} \times TIA_{area} + (n_{DDK\_cell} + n_{cell}) \times Cell_{area} = 4800 \times 0.068\, mm^2 + 3 \times 0.05\, mm^2 + 5578 \times 0.33\, mm^2 + (80 + 649600 \times 2) \times 6.25 \times 10^{-4}\, mm^2 = 2979.34\, mm^2 \quad \text{(S22)}$$

The maximal total energy consumption for the C3D network for UCF dataset could be estimated as:

$$E_{total} = n_{cell} \times E_{cell} + n_{DAC} \times E_{DAC} + n_{TIA} \times E_{TIA} + n_{ADC} \times E_{ADC} = 78025792 \times 2 \times 0.04\, pJ + 602112 \times 63\, pJ + 23390218 \times 2.88\, pJ + 3 \times 170\, pJ = 111539457.2\, pJ \quad \text{(S23)}$$

The area used could be estimated as:

$$Area_{total} = n_{DAC} \times DAC_{area} + n_{ADC} \times ADC_{area} + n_{TIA} \times TIA_{area} + n_{cell} \times Cell_{area} = 602112 \times 0.068\ mm^2 + 3 \times 0.05\ mm^2 + 23390218 \times 0.33\ mm^2 + 78025792 \times 2 \times 6.25 \times 10^{-4}\ mm^2 = 7857247.946\ mm^2 \quad \text{(S24)}$$

II: Supplementary Figures and Captions

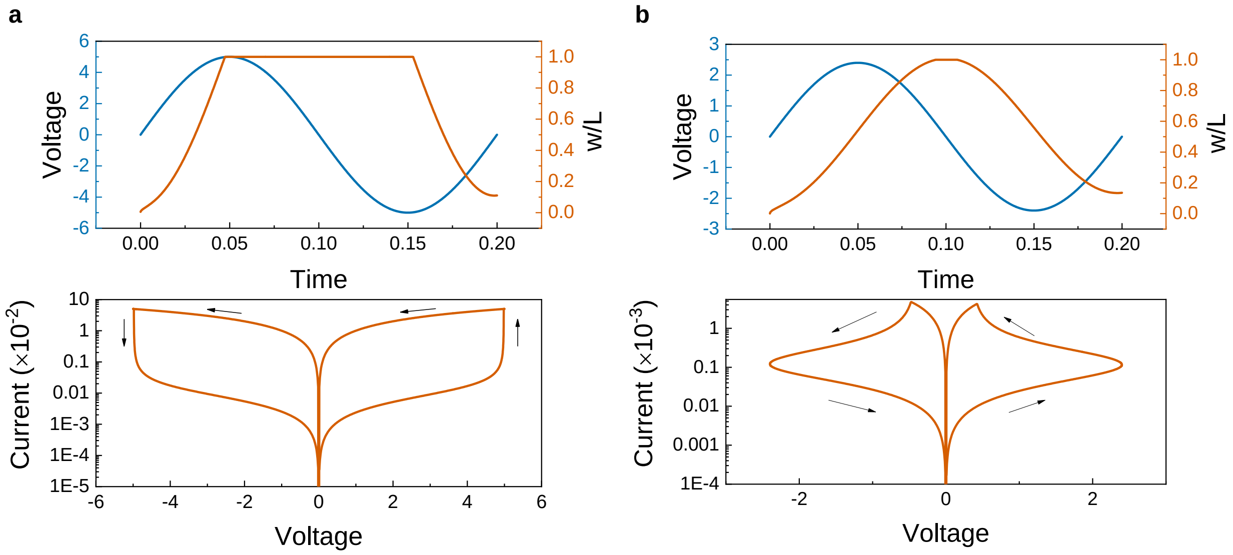

Fig. S1 | Typical RS curves obtained by the simplified drift-diffusion model. a, Abrupt RS. When the doped region arrives the boundary of the device, abrupt RS occurs. Typical parameters are $v_0 = 5$ V, $\omega_0 = 10$, $\mu = 6.3\times10^{-14}$ m^{2}V^{-1}s^{-1}, $R_{OFF}/R_{ON} = 450$ and $L = 10$ nm. **b,** NDR-type RS. When the doped region approaches the boundary of the device, NDR-type RS occurs. Typical parameters are $v_0 = 2.4$ V, $\omega_0 = 10$, $\mu = 6.3\times10^{-14}$ m^{2}V^{-1}s^{-1}, $R_{OFF}/R_{ON} = 450$ and $L = 10$ nm.

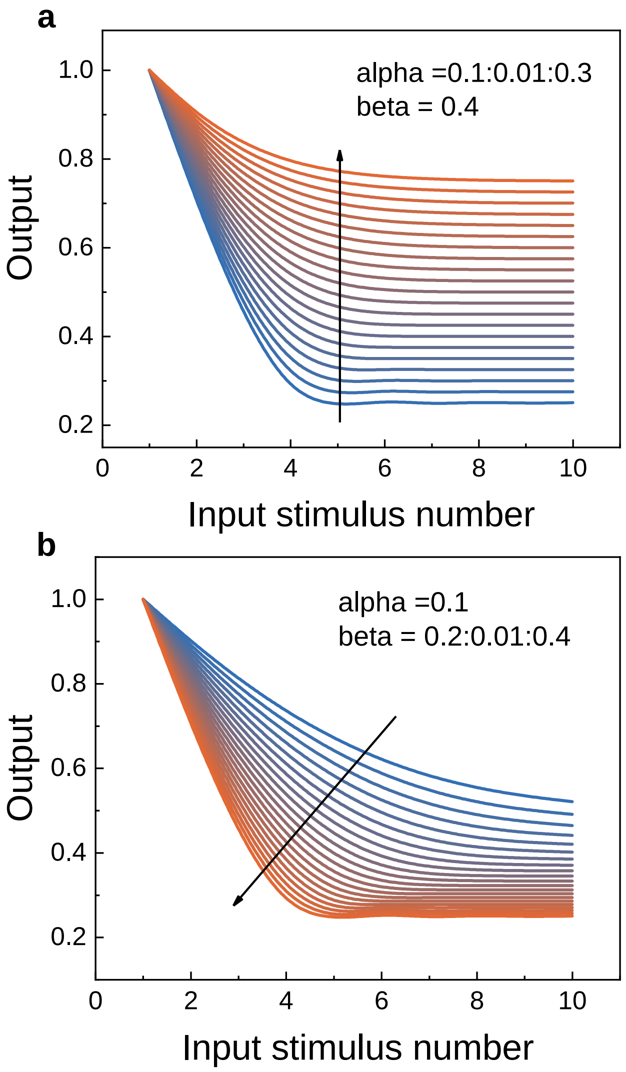

Fig. S2 | Influences of *α* and *β* on the output of the proposed model. a, Output with varying α (from 0.1 to 0.3 with a step of 0.01) and constant β (fixed at 0.4). With the increase of α, the asymptote of output also increases, indicating that α mainly influences the asymptote. **b,** Output with varying β (from 0.2 to 0.4 with a step of 0.01) and constant α (fixed at 0.1). With the increase of β, the output decreases more rapidly. Compared with α, β is more related to the decrease speed of output. However, the asymptote and evolution speed of output is determined by the combination of α and β.

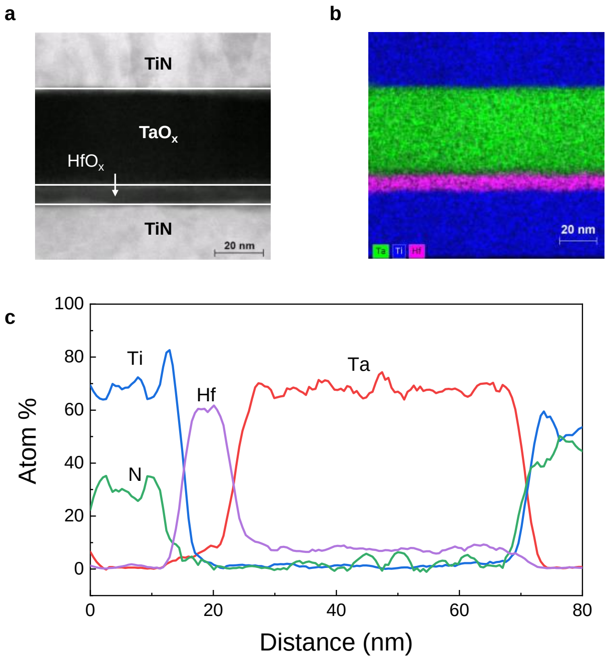

Fig. S3 | Structure and composition characterization of the TiN/TaO_x/HfO_x/TiN memristor device with HRTEM. a, The bilayer structure TaO_x/HfO_x sandwiched between two TiN electrodes. **b,** Area element distribution of Ta, Ti and Hf. The scale bars in **a** and **b** are 20 nm. **c,** EDS line scan in **b** shows clearly the device structure.

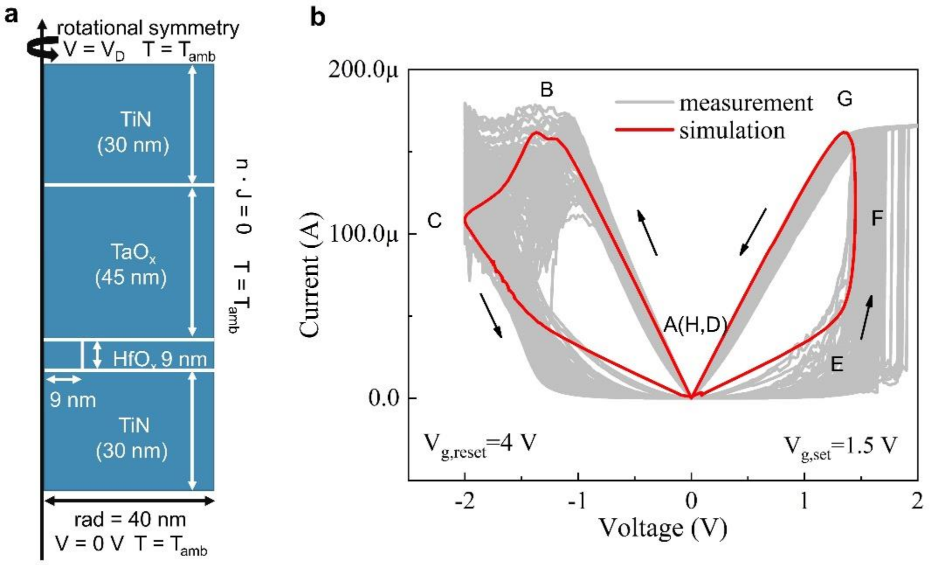

Fig. S4 | Finite-element modeling of the TiN/TaO_x/HfO_x/TiN device. a, Device geometry and boundary conditions. The device is modeled as a rotational symmetry cylinder. **b,** Simulated and measured *I-V* curves. The simulated results can well reproduce the RS of real devices.

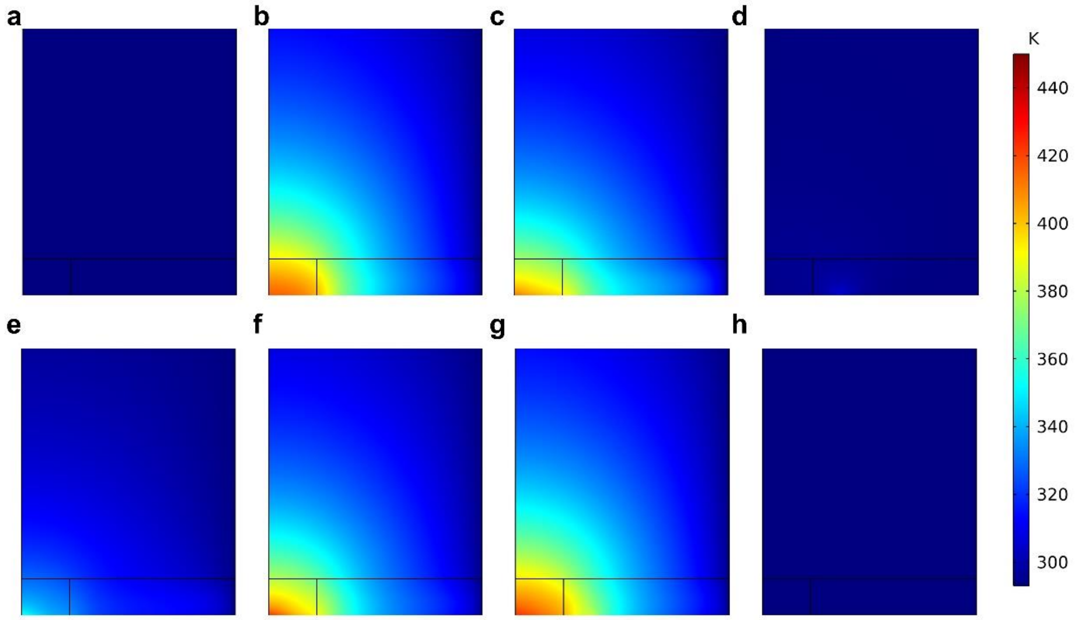

Fig. S5 | The temperature distribution for the TiN/TaO_x/HfO_x/TiN device during the reset (a-d) and set processes (e-h). The temperature distribution of **a,** at the initial stage (point A in Fig. S4b), **b,** near the reset voltage (point B in Fig. S4b), **c,** at the max negative voltage (point C in Fig. S4b), **d,** after completing the negative voltage scan (point D in Fig. S4b), **e,** near set voltage (point E in Fig. S4b), **f,** at the max voltage (point F in Fig. S4b), **g,** after set (point G in Fig. S4b), **h,** after completing the positive voltage scan.

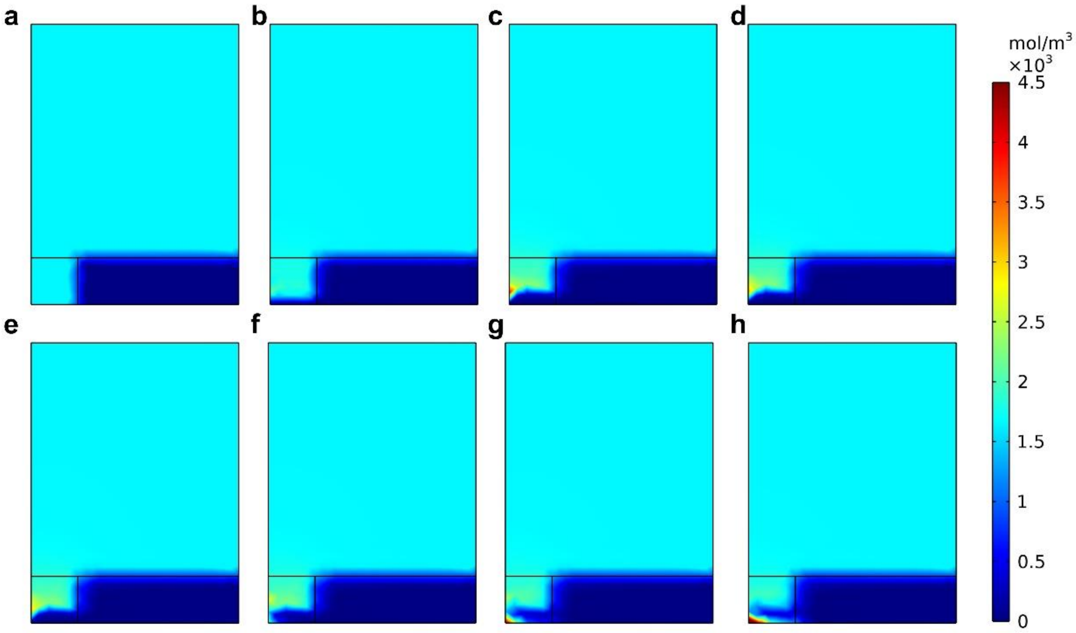

Fig. S6 | The oxygen vacancy distribution for the TiN/TaO_x/HfO_x/TiN device during the reset (a-d) and set processes (e-h). The oxygen vacancy distribution of **a,** at the initial stage (point A in Fig. S4b), **b,** at the edge of reset voltage (point B in Fig. S4b), **c,** at the max negative voltage (point C in Fig. S4b), **d,** after completing the negative voltage scan (point D in Fig. S4b), **e,** near set voltage (point E in Fig. S4b), **f,** at the edge of set voltage (point F in Fig. S4b), **g,** after set (point G in Fig. S4b), **h,** after completing the positive voltage scan.

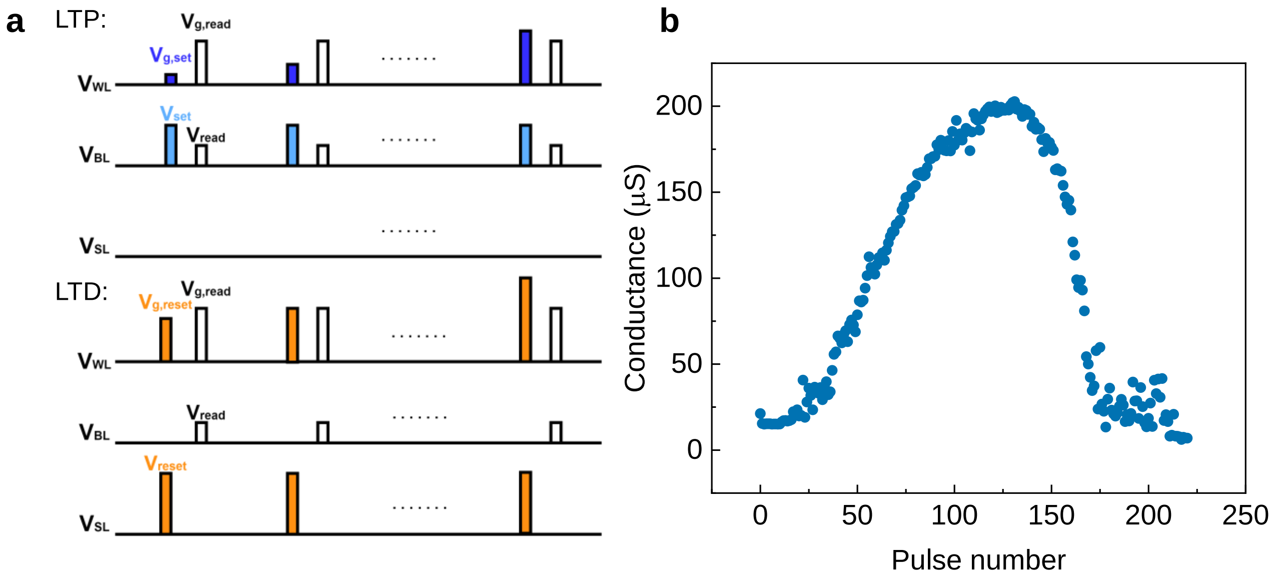

Fig. S7 | The pulsed *I-V-t* curves of the memristor. a, Programming scheme. For the LTP process, the voltage of the transistor gate (WL) increases linearly (from 0.9 V to 2.0 V), where the voltage of the top electrode is fixed at 2.2 V and the voltage of the source is ground. For the LTD process, each operation includes a SET and RESET pulse. For each operation, the RESET pulse is applied first, then the SET pulse is applied. The RESET voltages of the gate, source, and top electrode are fixed (3.7 V, ground, and -3.3 V separately). The SET voltage of the gate decreases linearly (from 2.0 V to 0.9 V). The SET voltage of the top electrode is fixed at 2.2V, while the source is grounded. The pulse width in the measurement is fixed at 5 ns. The conductance is read at 0.2 V. **b,** A typical LTP/LTD curve of the memristor device.

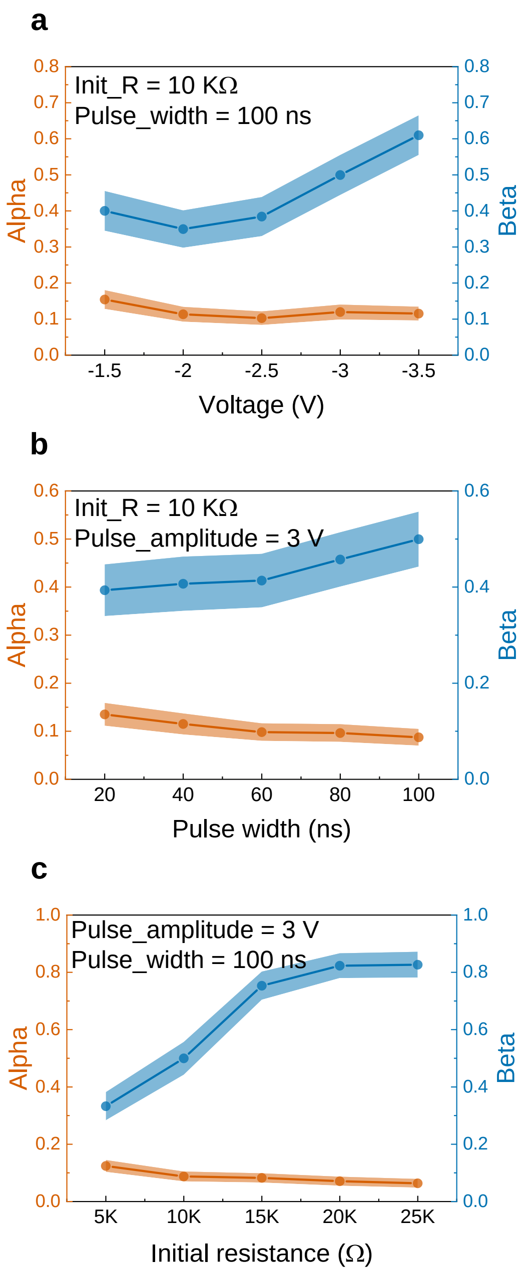

Fig. S8 | Device-to-device test for tunability of parameters *α* and *β*. a, Parameter variation with voltage pulse amplitude. The initial resistance and pulse width are fixed at 10 KΩ and 100 ns, respectively. At small voltages (–1.5 and –2 V), *β* does not show strong dependence on voltage amplitude probably since the voltage pulse is too weak to induce significant RS. However, with large voltage pulses, a clear dependence of *β* on pulse amplitude is observed. **b,** Parameter variation with voltage pulse width. A positive correlation between pulse width and *β* is shown. **c,** Parameter variation with initial resistance states. With high initial resistance, *β* becomes large and there is an asymptote. Statistics of *α* and *β* are presented in error band diagrams (95% confident interval).

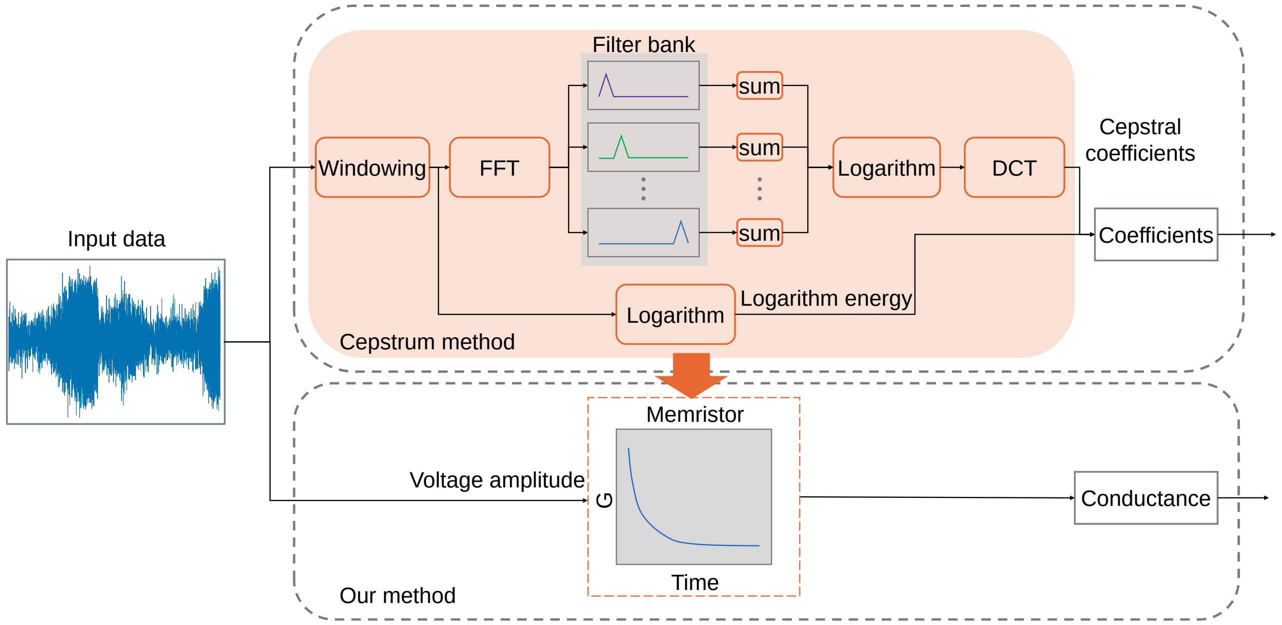

Fig. S9 | Comparison of cepstrum method and the proposed method in 1-D raw data processing. Cepstrum methods are widely used feature extraction techniques in state-of-the-art classification tasks of 1-D temporal data, e.g., speech or speaker recognition, electroencephalogram (EEG) recognition and so on. Mel filter bank is usually used in cepstrum method to extract MFCC. The procedure of obtaining MFCC includes short-time Fourier transformation (STFT), Mel filter bank, discrete cosine transformation (DCT) and so on, which is time- and energy consuming. In contrast, our method is more straightforward and simpler. The feature extraction is completed within a single memristor.

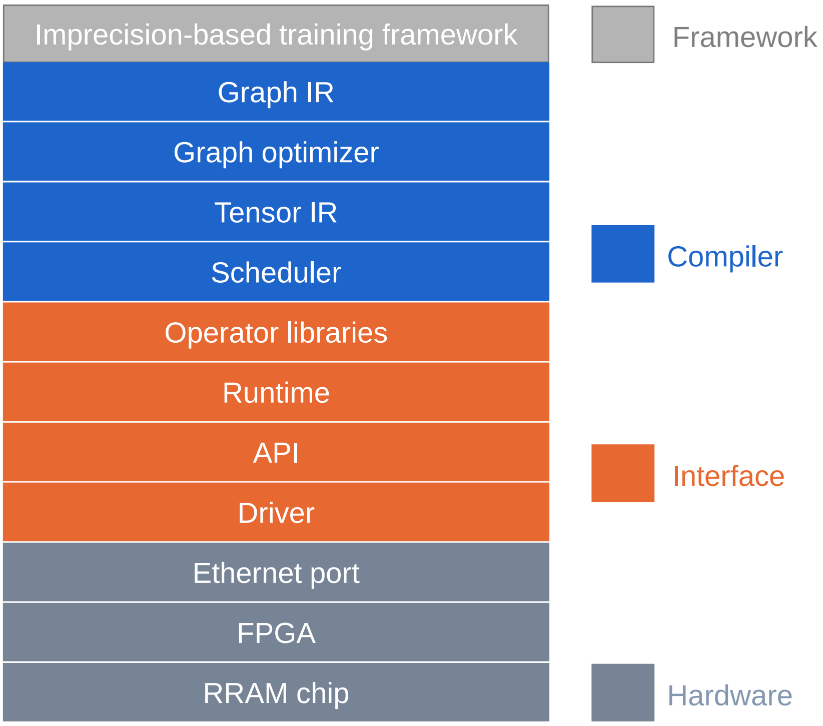

Fig. S10 | Architecture of a hardware-software system for deployment. The software part includes an imprecision-based neural network training framework and a compiler. The hardware part is a printed circuit board (PCB)-based test system. An interface connects the software and hardware parts. IR: intermediate representation.

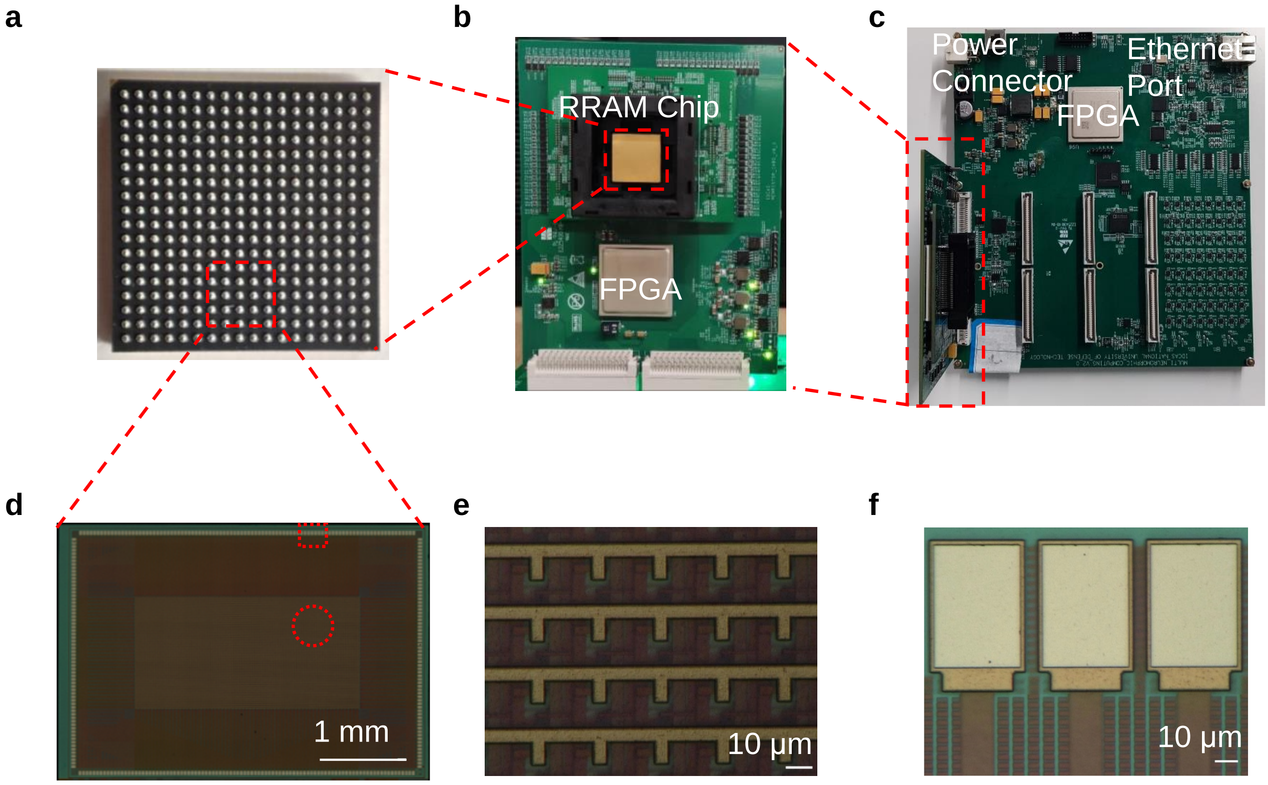

Fig. S11 | Memristor-based hardware system. a, A ceramic ball grid array (BGA) package for the chip. **b,** Sub-PCB for chip setup and configuration. **c,** Main PCB for chip measurement, power management, communication, and configuration. **d,** Micrograph of an 8 Kb crossbar array. **e,** Zoomed-in micrograph of an array region circled in d. **f,** Zoomed-in micrograph of the pads in the rectangle region in d.

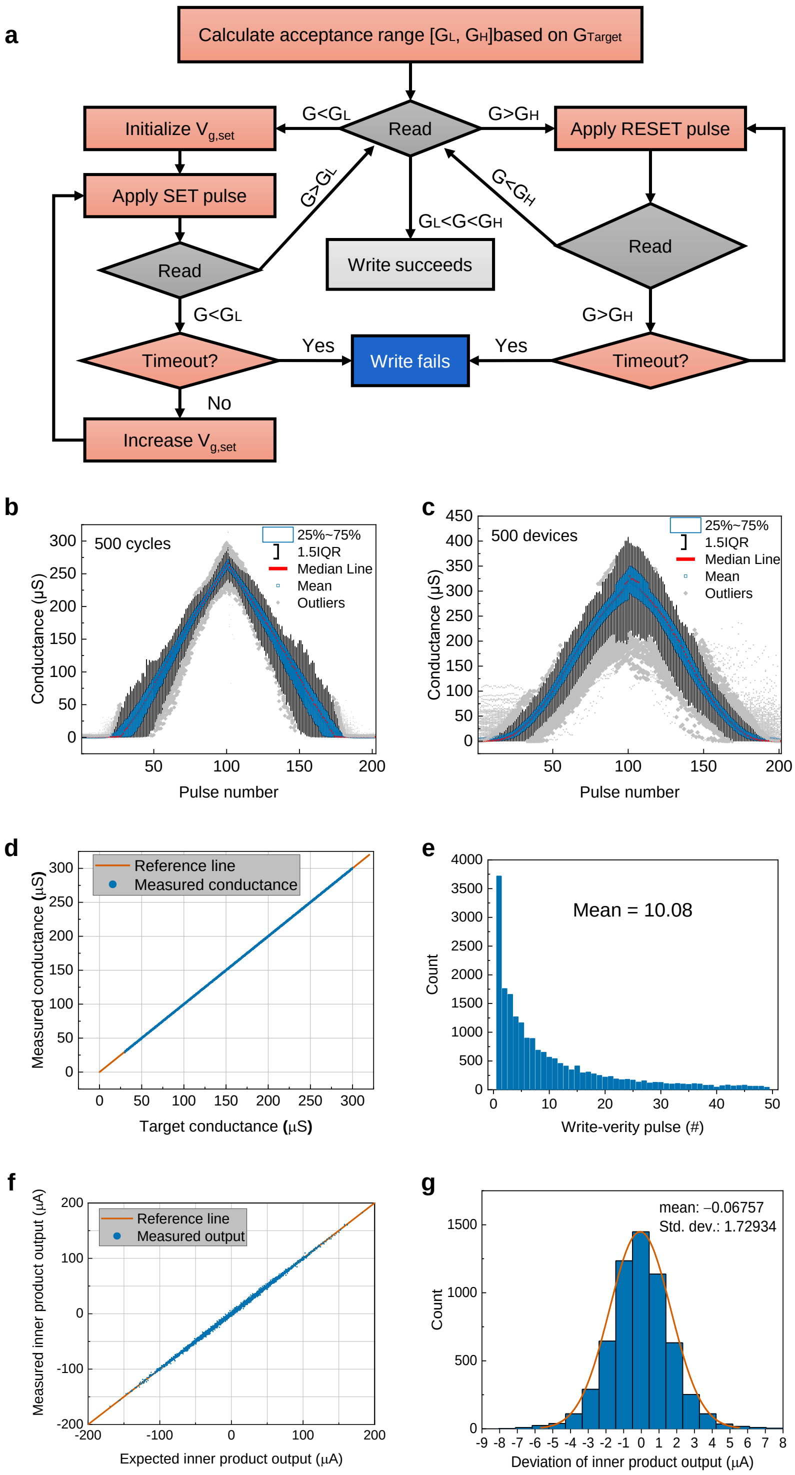

Fig. S12 | Device programming and MVM output. a, Flow chart of write-and-verify technique for programming device conductance to a desired value. **b,** Boxplot of long-term potentiation and depression (LTP/LTD) test of a single memristor for 500 cycles, the conductance change curve shows high linearity and symmetry. **c,** Boxplot of LTP/LTD test of 500 memristors. **d,** Actually measured and ideal target conductance in 30 K weight updating operations. **e,** Distribution of the number of pulses needed for the device to be programmed to the desired conductance value. **f,** Measured and expected MVM output in 6 K MVM operations. **g,** Statistics of the deviation of measured MVM output from expected one.

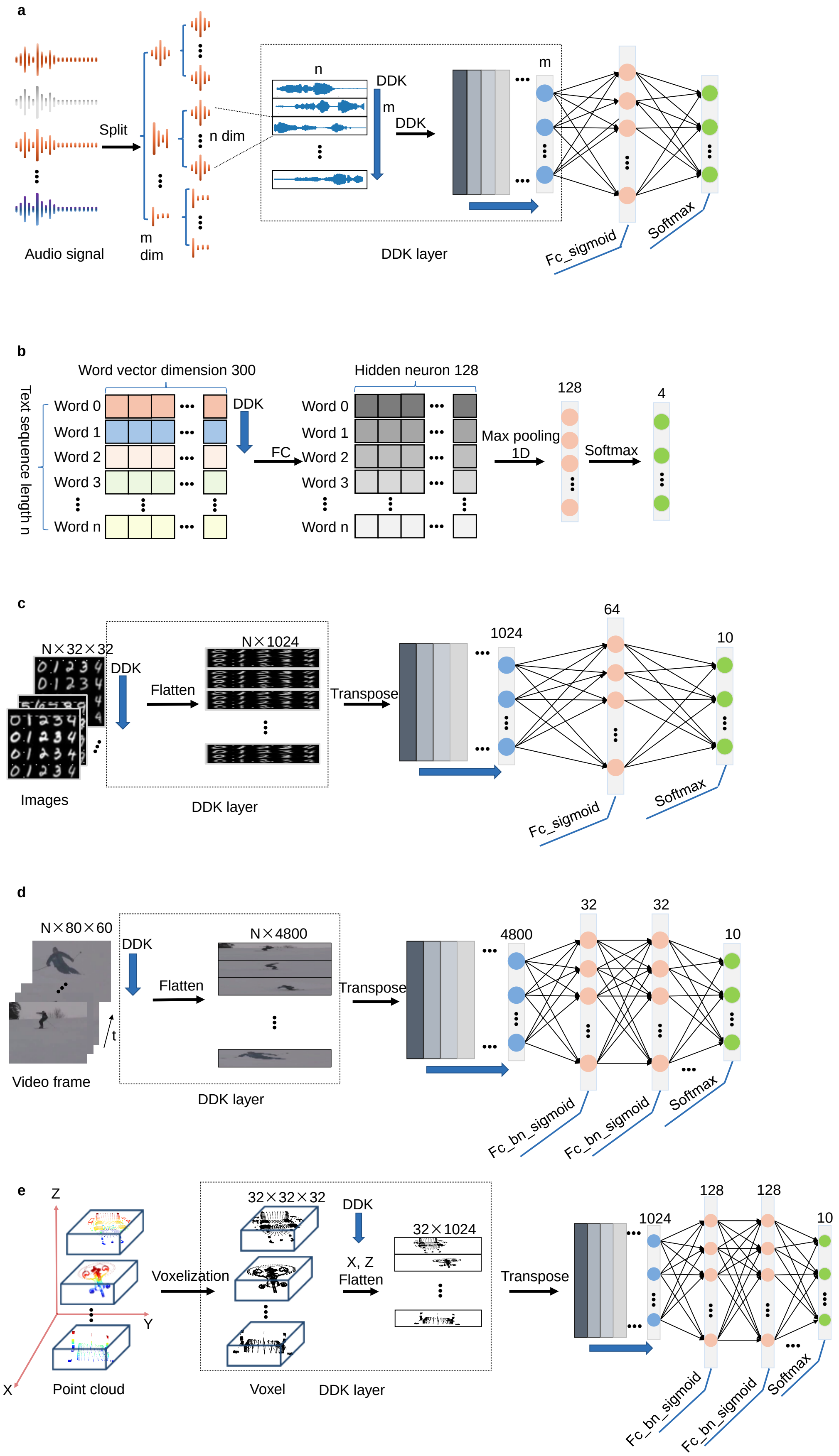

Fig. S13 | DDK neural network architecture. a, Raw waveform classification. **b**, Text classification. **c**, Image classification. **d**, Video stream classification. **e**, 3-D object classification.

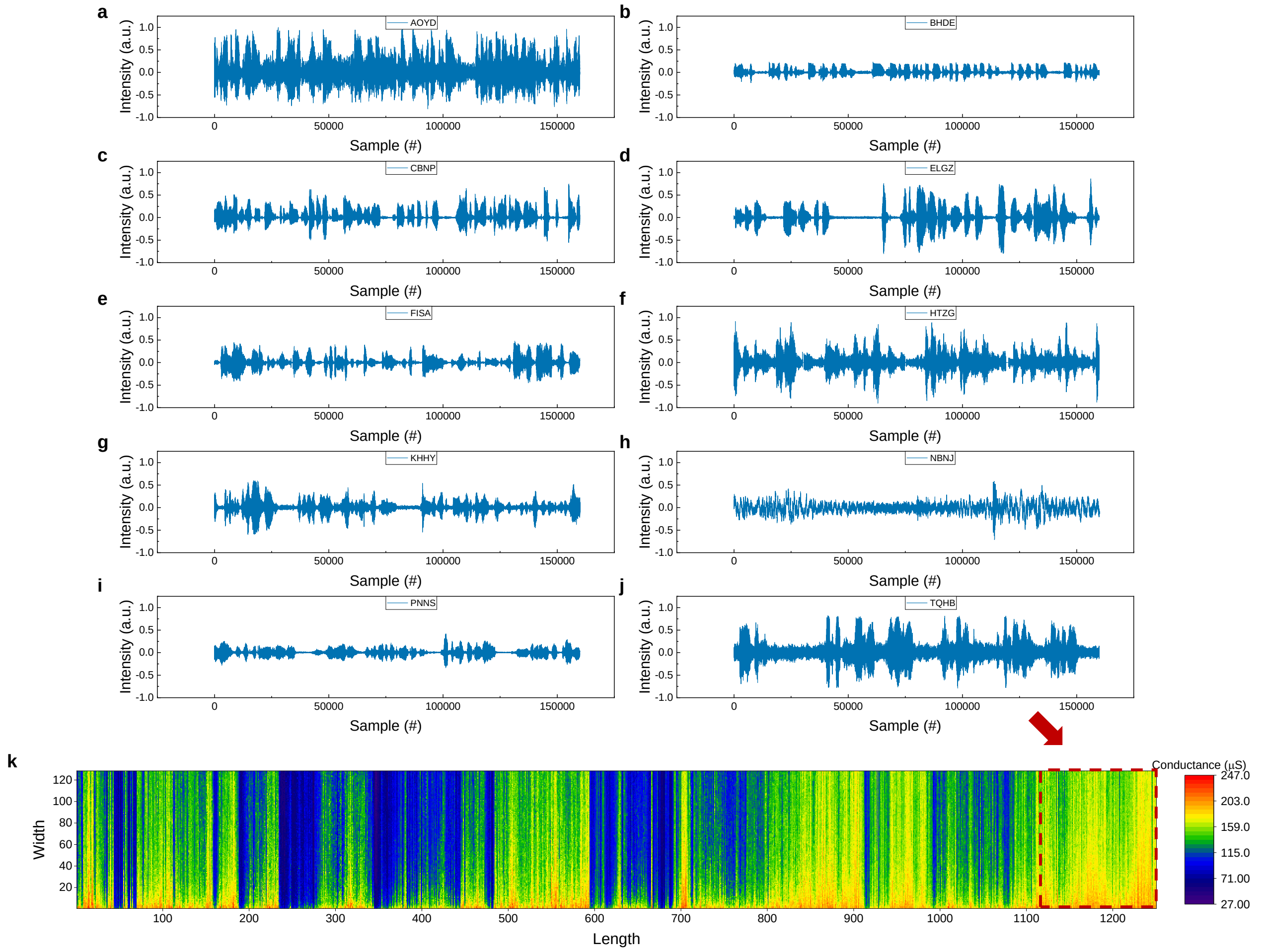

Fig. S14 | Feature maps experimentally obtained with memristors. a-j, 10 Raw audio waveform clips of 10 speakers in SITW dataset. **k,** The concatenated DDK feature maps of the 10 waveform clips experimentally obtained by memristors.

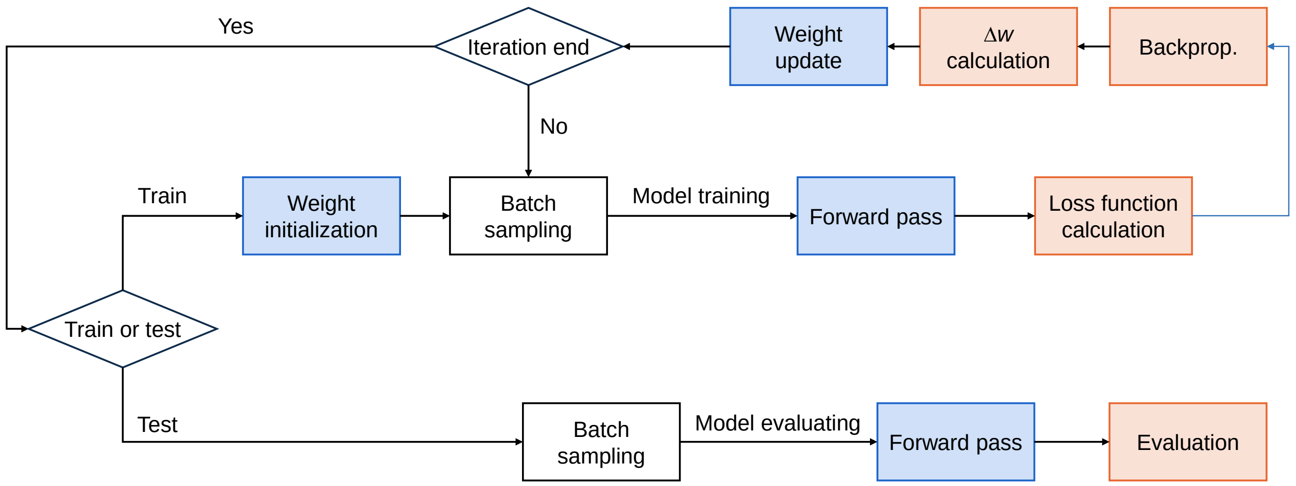

Fig. S15 | *In-situ* training flowchart. Flow chart for *in-situ* training of the memristive neural network. The blue blocks are implemented with memristive hardware, while the orange blocks are realized with CPU.

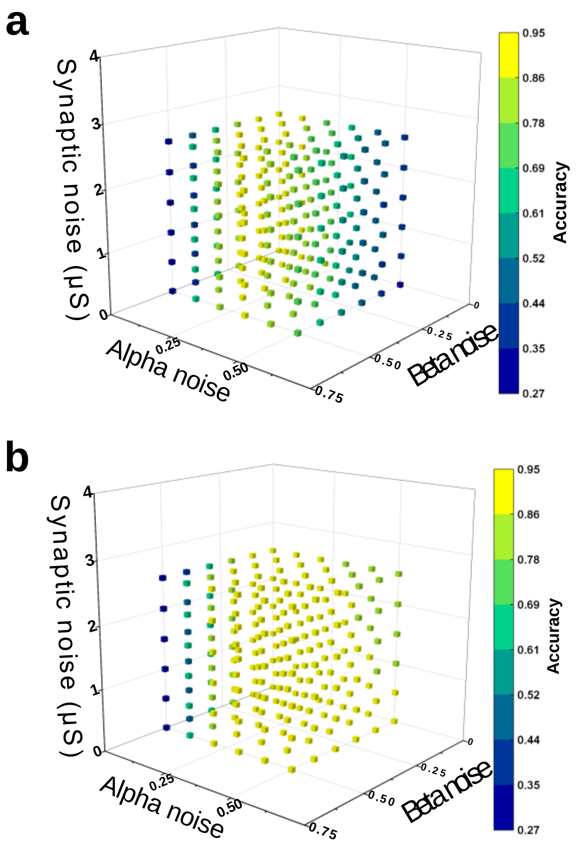

Fig. S16 | Influence of cycle-to-cycle variation on DDK network in audio classification. a, The impact of α noise, β noise, and synaptic noise on the classification accuracy of DDK network on the SITW dataset (deployed directly after off-chip training). **b**, The impact of α noise, β noise, and synaptic noise on the classification accuracy of DDK network on the SITW dataset when α and β are fixed, and synaptic weights are fine-tuned. The classification accuracy gradually decreases as the noise increases. After fine-tuning the synaptic weights, the impact of α noise, β noise, and synaptic noise on the classification accuracy is significantly reduced.

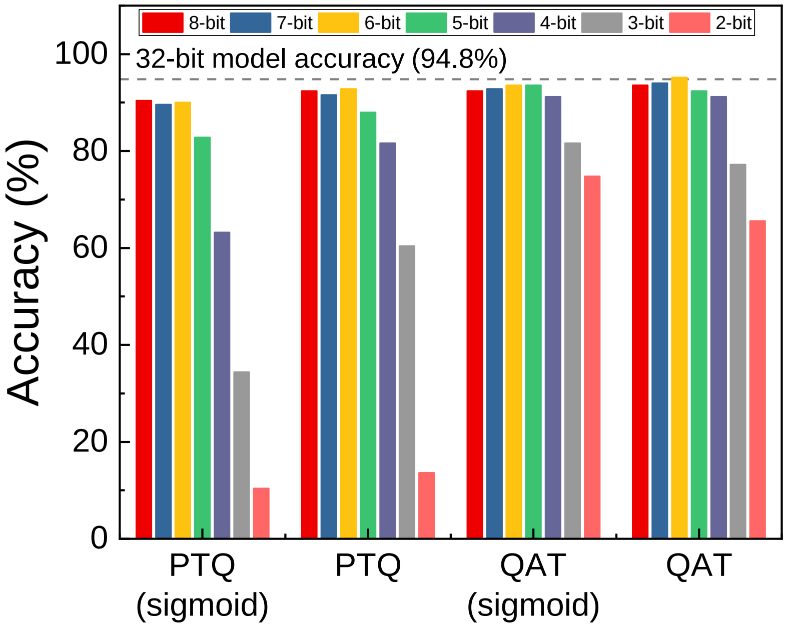

Fig. S17 | Quantization performances of the DDK network on SITW dataset. The accuracy refers to the maximal accuracy in 5 simulations. The quantization methods are as follows. PTQ (sigmoid): post-training quantization with sigmoid activation function quantization; PTQ: post-training quantization without sigmoid activation function quantization; QAT (sigmoid): quantization-aware training with sigmoid activation function quantization; QAT: quantization-aware training without sigmoid activation function quantization. The overall accuracy loss of QAT is less than PTQ. For PTQ, sigmoid activation function quantization is not preferred, while for QAT, sigmoid activation function quantization can usually lead to better performance. The dashed line shows the accuracy of a floating point 32-bit model without quantization.

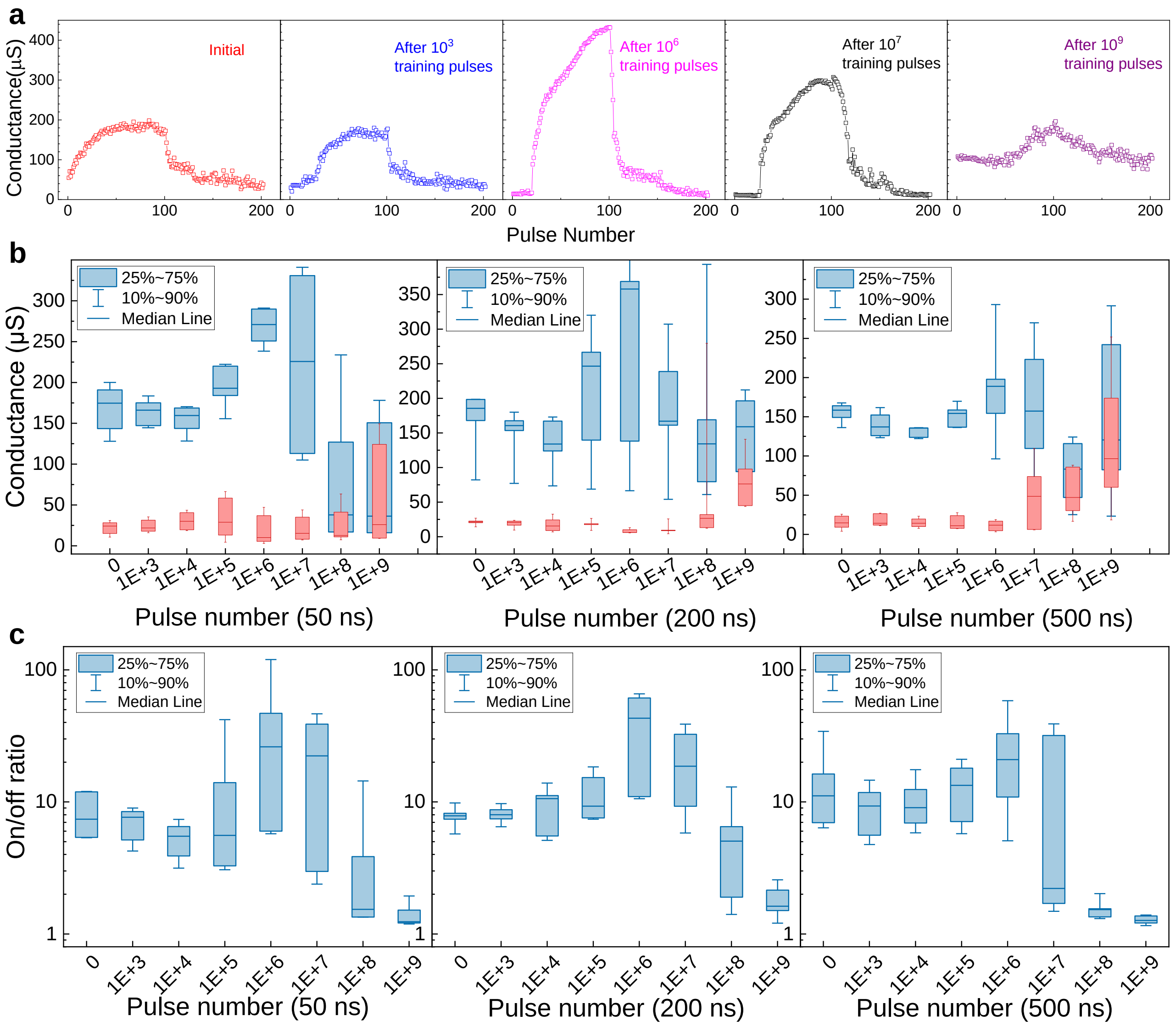

Fig. S18 | Endurance test of the memristor device. **a,** Analog switching of the full dynamic range of different operation numbers (pulse width is 200 ns). **b,** Achievable conductance range for different pulses with width of 50 ns, 200 ns and 500 ns. The maximum and minimum conductance of each cycle are collected to obtain the conductance range. We collect the analog switching of at least 5 devices for each pulse width configuration and display the boxplot of the conductance range, which shows that the achievable conductance range is degraded as the pulse number increases. **c,** On/off ratio of the at least 5 devices after different numbers of update pulses, the on/off ratio can still be larger than 1 after 10^9 incremental switching.

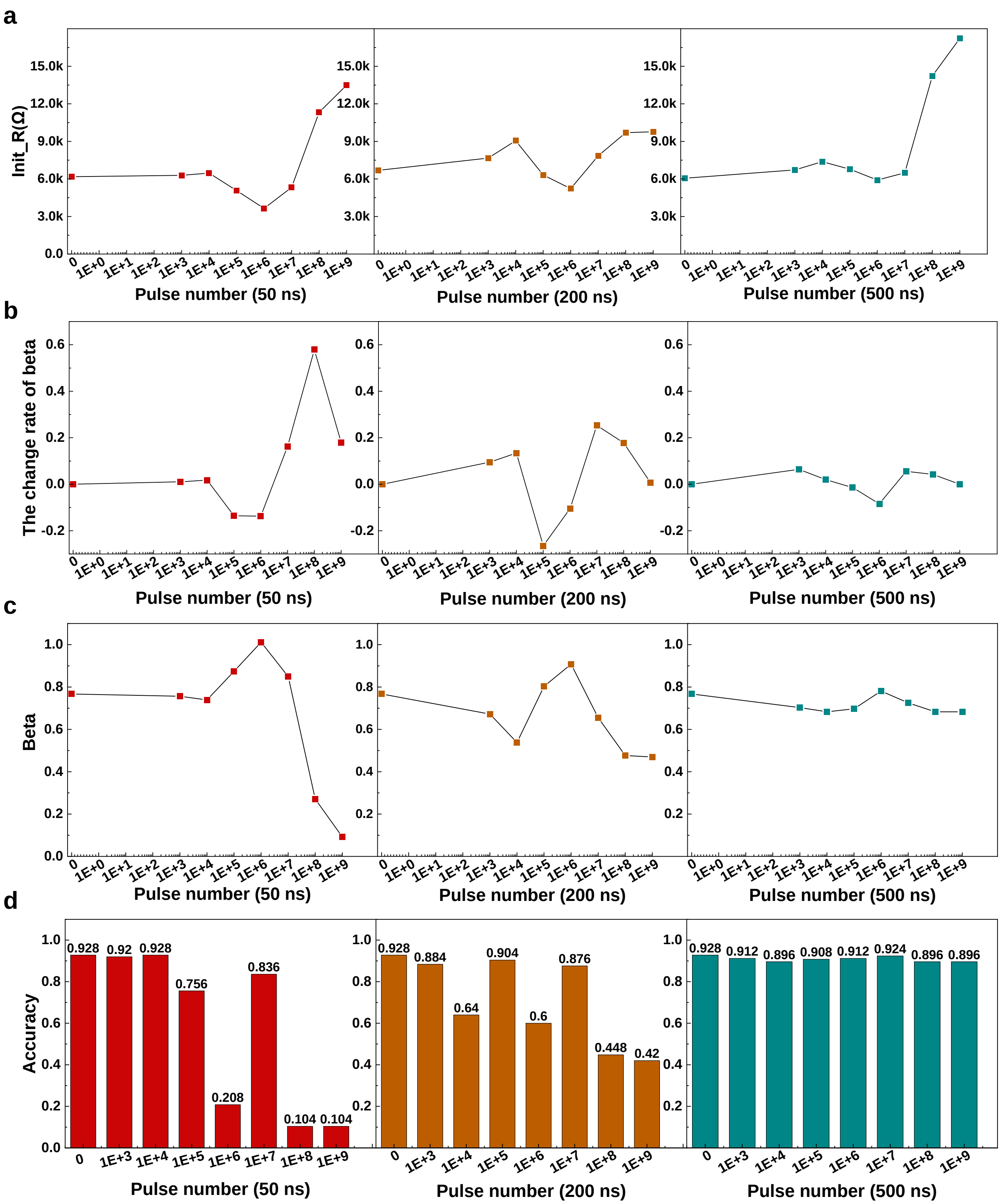

Fig. S19 | The relation of latency and accuracy in the endurance test in audio classification task. **a.** Changes in initial resistance at different pulse widths. The values are average high conductance states of at least 5 test devices for each pulse configuration. **b**. The change rate of β at different pulse widths. **c**. The decay process of β from the pre-trained network at different pulse widths. **d**. The effect of β decay on classification accuracy in audio classification at different pulse widths.

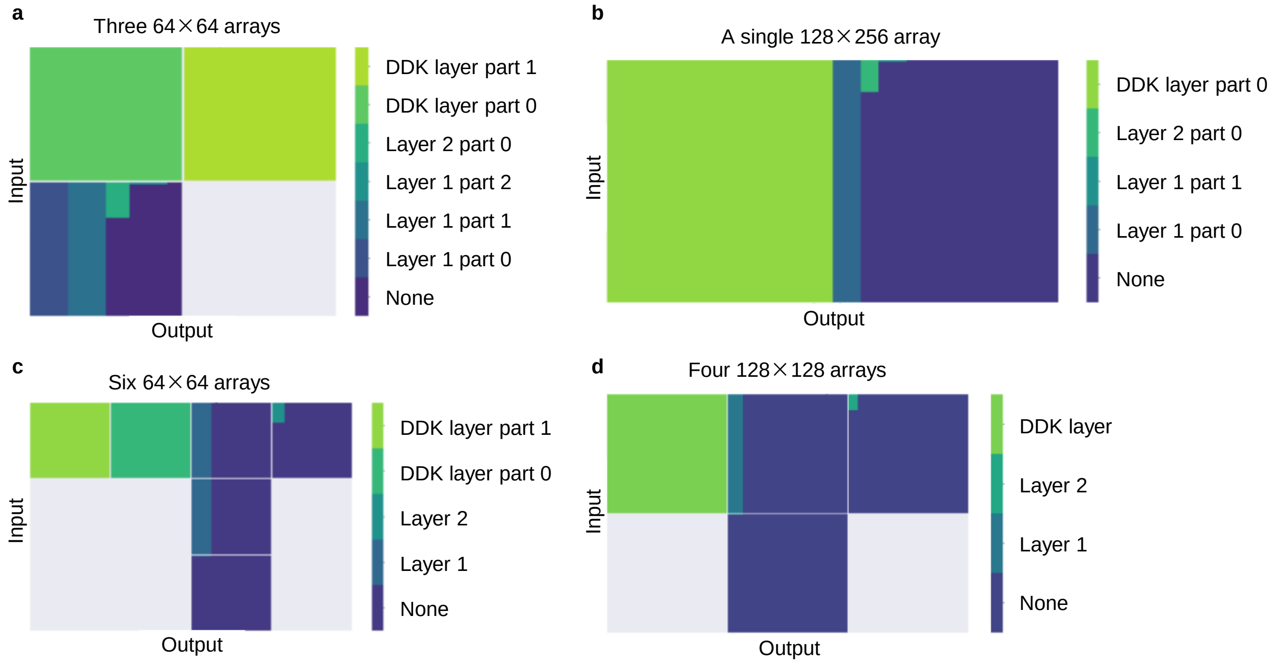

Fig. S20 | Comparison of compiler-generated mapping strategies. a, Strategy A, three 64×64 arrays. **b,** Strategy B, a single 128×256 array. **c,** Strategy C, six 64×64 arrays. **d,** Strategy D, four 128×128 arrays.

III: Supplementary Tables

Table S1 | Material parameters in finite-element simulation

Symbol	Value	Description
a[21]	0.1 [nm]	Hopping distance
f[22]	1×10^{12} [Hz]	Escape-attempt frequency
$E_{a\_TaO_x}$[21]	0.68 [eV]	Diffusion activation energy of TaO_x
T_0	293.15 [K]	Initial temperature
λ[21]	0.1 [1/K]	Thermal coefficient
k_{HfO_x}[23]	0.5 [W/(m•K)]	Thermal conductivity of HfO_x for $n_{V_O^{\cdot\cdot}}$ = 0 cm^{-3}
k_{TaO_x}[24]	3 [W/(m•K)]	Thermal conductivity of TaO_x at T_0 for $n_{V_O^{\cdot\cdot}}$ = 1 × 10^{21} cm^{-3}
k_electrode	5 [W/(m•K)]	Electrode thermal conductivity of TiN
$E_{a\_HfO_x}$[25]	0.65 [eV]	Diffusion activation energy of HfO_x
$E_{ac_{TaO_x}}$[21]	–0.006 [eV]	Activation energy for conduction of TaO_x for $n_{V_O^{\cdot\cdot}}$ = 1 × 10^{21} cm^{-3}
$E_{ac_{HfO_x}}$[26]	0.05 [eV]	Activation energy for conduction of HfO_x for $n_{V_O^{\cdot\cdot}}$ = 0 cm^{-3}
σ_{TaO_x}[21]	9.4×10^4 [S/m]	Electrical conductivity of TaO_x for $n_{V_O^{\cdot\cdot}}$ = 1 × 10^{21} cm^{-3}
σ_{HfO_x}[26,27]	3 [S/m]	Electrical conductivity of HfO_x for $n_{V_O^{\cdot\cdot}}$ = 0
$\sigma_{electrode}$	3×10^4 [S/m]	Electrical conductivity of TiN

Notes:

Diffusion activation energy(E_a): In general, the E_a of HfO_2 is conventionally considered to be 0.7 eV[25]. The E_a of TaO_x has been reported as 0.85 eV in some literature[21]. To achieve the best fitting results between simulation data and our experimental results, we set the E_a of HfO_x ($E_{a\_HfO_x}$) to 0.65 eV and that of TaO_x ($E_{a\_TaO_x}$) to 0.68 eV, values which are close to those previously reported [21,25].

Thermal conductivity:

According to literature reports, the thermal conductivity of TaO_x (k_{TaO_x}) thin films varies between 0.9 W/m·K (low $n_{V_O^{\cdot\cdot}}$) and 4 W/m·K (high $n_{V_O^{\cdot\cdot}}$)[24]. In this study, we choose a thermal conductivity value of 3 W/m·K for TaO_x at $n_{V_O^{\cdot\cdot}} = 1 \times 10^{21}$ cm^{-3}, which is consistent with the reported values in the literature[24].

Electrical conductivity: HfO_2 is commonly used as a high-k gate insulator in microelectronic devices, and its electrical conductivity is influenced by various factors. According to previous reports, the electrical conductivity of HfO_2 is extremely low when the $n_{V_O^{\cdot\cdot}}$ is zero[26,27]. In this study, we set the electrical conductivity of HfO_x to 3 S/m for $n_{V_O^{\cdot\cdot}} = 0$ cm^{-3}, which is consistent with prior research reporting values ranging from 0 to 1000 S/m[26,27].

Table S2 | Comparison of various hardware for MFCC feature extraction

	Tech. node	Name	Frequency	Power	Latency	Energy
This work	180 nm	N.A.	100 MHz	80 μW	10 ns	0.8 pJ
ASIC[1]	28 nm	N.A.	0.04 MHz	0.34 μW	16 ms	5.44 nJ
ASIC[2]	65 nm	N.A.	0.25 MHz	21.23 μW	2 ms	42.46 nJ
ASIC[3]	180 nm	N.A.	N.A.	N.A.	45.79 μs	0.72 μJ
FPGA[4]	90 nm	XC4VLX15	50 MHz	172 mW	292 μs	50.22μJ
FPGA[5]	90 nm	XC3S2000	50 MHz	234 mW	285.44 μs	66.79 μJ
GPU[6]	40 nm	GTX580	772 MHz(Core)	244 W	9.17 μs	2.24 mJ
DSP[8]	130 nm	TMS320C6713	225 MHz	1.067 W	0.912 ms	0.973 mJ
CPU[5]	180 nm	Intel P4	1.5 GHz	57.8 W	238.77 μs	13.8 mJ

Table S3 | Training hyperparameters of DDK neural networks

	SITW	AGNews	MNIST	UCF	ModelNet	Notes
Learning rate	0.01	0.001	0.01	0.01	0.01	The adjustment in weights with respect to loss gradient
MFL_dim	2	2	2	120	64	The number of MFL layer parameters
Hidden_dim	16	128	64	128	128	The number of channels of the hidden layer
Num_fc_sigmoid	1		1			The number of hidden layers consisting of fully connected, batch normalization and sigmoid activation function
Num_fc_bn_sigmoid				3	3	The number of hidden layers consisting of fully connected, batch normalization and ReLU activation function
Num_fc_tanh		1				The number of hidden layers consisting of fully connected and tanh activation function
Maxpooling_1D		64				The size of max pooling 1D
Output_dim	10	4	10	10	10	The number of channels of the output layer
Num_epoch	500	20	50	500	500	The number of iteration

Table S4 | Training hyperparameters and physical parameters of memristor array

Parameter	Value	Description
β_1	0.9	Coefficients for computing running averages of gradient
β_2	0.999	Coefficients for computing running averages of gradient square
ε	1e-8	Denominator shift for Adam
G/W	8e-5	Conductance-to-weight ratio
$\Delta G/\Delta W$	8e-5	Weight gradient-to-conductance gradient ratio
V_{set}	2.4	Set voltage
V_{reset}	2.5	Reset voltage
V_{read}	0.2	Read voltage
$V_{gate,reset}$	3.7	Reset gate voltage
$V_{gate,max}$	2.1	Maximum set gate voltage
$V_{gate,min}$	1.1	Minimum set gate voltage

Table S5 | Training hyperparameters of convolutional and MFCC-based neural networks

	SITW	SITW	Notes
Network	CNN	MFCC	Network structure for classification
Learning rate	0.01	0.01	The adjustment in weights with respect to loss gradient
N_Mel		40	The number of Mel filters
N_conv	5		The number of convolution layers
Conv1_filter_size	(128,1,3,3)		The 1st convolution kernel:(output channel, input channel, kernel size, stride)
Conv2_filter_size	(128,128,3,1)		The 2nd convolution kernel:(output channel, input channel, kernel size, stride)
Conv3_filter_size	(128,128,3,1)		The 3rd convolution kernel:(output channel, input channel, kernel size, stride)
Conv4_filter_size	(256,128,3,1)		The 4th convolution kernel:(output channel, input channel, kernel size, stride)
Conv5_filter_size	(256,256,3,1)		The 5th convolution kernel:(output channel, input channel, kernel size, stride)
Max pooling_size	3		The size of max pooling
N_conv_bn_relu_max pooling	5		The number of modules consisting of convolution, batch normalization , ReLU and max pooling
Fc1_dim	512		The number of channels of the 1st fully connected layer
N_hidden_layer		3	The number of hidden layers
Hidden_dim		32	The number of channels of the hidden layer
Output_dim	10	10	The number of channels of the output layer
N_epoch	500	500	The number of iteration

Table S6 | Comparison of DDK network with other DNNs in terms of operation numbers (MACs) in feature extraction and classification

Dataset	Network	MACs/ feature extraction	MACs/ classifier	MACs/ Total	Proportion of MACs in the feature extraction
SITW	DDK	256	2.208K	2.464K	0.1039
	SL-CNN	15.322M	0.267M	15.589M	0.9829
AGNews	DDK	38.4K	2.459M	2.497M	0.0154
	Transformer	1.5749G	76.8K	1.575G	0.9999
	LSTM	257.294M	2048	257.296M	0.9999
MNIST	DDK	1568	38.17K	39.738K	0.0395
	LeNet	141.048K	58.92K	199.968K	0.705
UCF	DDK	9.6K	649.984K	659.584K	0.0146
	C3D	38.497G	0.05G	38.547G	0.9987
ModelNet	DDK	2048	166.656K	168.704K	0.012
	VoxNet	58.752M	0.886M	59.638M	0.9851

Table S7 | The number of parameters and operations of MFCC

MFCC	#muls	#add	#parameter
Pre-emphasis	N	N	1
Windowing	N	-	N
FFT	$2N\log_2 N$	$6N\log_2 N$	-
Mel filtering	2N	2N	M(N/2+1)
DCT	MF	MF	-

M: number of MFCC features; N: FFT size; F: number of Mel filters.

Table S8 | Comparison of various layer types based on computational efficiency metrics (per-layer complexity, minimum number of sequential operations and maximum path length).

Layer type	Complexity per layer	Sequential operations	Maximum path length
This work (DDK)	$O(n \cdot d)$	$O(n)$	$O(1)$
Self-attention	$O(n^2 \cdot d)$	$O(1)$	$O(1)$
Recurrent	$O(n \cdot d^2)$	$O(n)$	$O(n)$
Convolutional	$O(k \cdot n \cdot d^2)$	$O(1)$	$O(\log_k(n))$
Fully connected	$O(n^2 \cdot d^2)$	$O(1)$	$O(1)$

n: sequence length; d: representation dimension; k: kernel size of convolutions

Table S9 | Hardware parameters at 180 nm technology node in simulation

Circuit	TIA[28]	ADC[29]	DAC[30]
Technology	180 nm	180 nm	180 nm
Resolution (bit)	8	8	8
Power (mW)	7.2	8.5	12.6
Frequency (MHz)	2500	200	200
Area (mm^2)	0.33	0.05	0.068
Voltage (V)	1.8	1.8	1.8
Current (mA)	4	4.72	7

Table S10 | Detailed metrics of each circuit module of the memristive hardware system in the mapping strategies

Mapping		Strategy A	Strategy B	Strategy C	Strategy D
Number of ADCs		16	32	96	128
Number of DACs		16	32	96	128
Number of TIAs		2	3	12	12
Area (mm^2)	ADC	0.8	1.6	4.8	6.4
	DAC	1.09	2.18	6.53	8.704
	TIA	0.66	0.99	3.96	3.96
	Array	0.68	1.81	1.36	3.62
	Sum	3.23	6.59	16.65	22.68
Energy (pJ)	ADC	7905	7225	7905	7225
	DAC	17262	17262	17262	17262
	TIA	535.68	489.6	535.68	489.6
	Array	166.82	297.89	166.82	297.89
	Sum	25869.5	25274.5	25869.5	25274.5
Latency (cycles)		6	4	1	1
Latency (ns)		120	80	20	20

Table S11 | Benchmark of the mapping strategies

	Strategy A	Strategy B	Strategy C	Strategy D
Operation (ops)	70004			
Performance ($GOP\ s^{-1}$)	583.37	875.05	3500.2	3500.2
Power (mW)	215.58	315.93	1293.47	1263.72
Area (mm^2)	3.23	6.59	16.65	22.68
Energy efficiency ($TOP\ s^{-1}\ W^{-1}$)	2.71	2.77	2.71	2.77
Performance density ($GOP\ s^{-1}\ mm^{-2}$, INT8)	180.79	133.07	210.28	154.3

Table S12 | The number of operations of the DDK network for SITW

Layer	Input Neurons	Output Neurons	Number of weights	Number of DDK operation
DDK	$n_{input\_dim} = 128$	128	2	128
FC1	128	16	128×16+16	-
FC	16	$n_{output\_dim} = 10$	16×10+10	-
Total		$n_{output\_neurons} = 154$	$n_{mlp\_weights} = 2234$	
Total number of devices		$n_{mlp_{devices}} + n_{DDK_{devices}}$<br>$= 2234 \times 2 + 1 = 4469$		

Table S13 | The number of operations of the CNN for SITW

Layer	Input Neurons	Output Neurons	Number of weights
conv1	$n_{input\_dim} = 1600$	128×534	128×1×3
BN1	128×534	128×534	256
conv2	128×178	128×178	128×128×3
BN2	128×178	128×178	256
conv3	128×59	128×59	128×128×3
BN3	128×59	128×59	256
conv4	128×19	256×19	256×128×3
BN4	256×19	256×19	512
conv5	256×6	256×6	256×256×3
BN5	256×6	256×6	512
fc1	256×2	512	512×512
fc	512	$n_{output\_dim} = 10$	512×10
Total		$n_{\mathrm{output\_}neurons} = 210698$	$n_{weights} = 662656$
Total number of devices	662656×2=1325312		

Table S14 | The number of operations of the DDK network for UCF

Layer	Input Neurons	Output Neurons	Number of weights	Number of DDK operation
DDK	$n_{input\_dim} = 4800$	4800	120	4800
FC1	4800	128	4800×128+128	-
BN1	128	128	256	-
FC2	128	128	128×128+128	-
BN2	128	128	256	-
FC3	128	128	128×128+128	
BN3	128	128	256	-
FC4	128	$n_{output\_dim} = 10$	128×10	-
Total		$n_{output\_neuron} = 5578$	$n_{mlp\_weight} = 649600$	
Total number of devices	$n_{mlp\_device} + n_{DDK\_device} = 649600 \times 2 + 80 = 1299280$			

Table S15 | The number of operations of the CNN for UCF

Layer	Input Neurons	Output Neurons	Number of weights
conv1	$n_{input\_dim} = 3 \times 16 \times 112 \times 112$	64×16×112×112	64×3×3×3×3
conv2	64×16×56×56	128×16×56×56	128×64×3×3×3
conv3a	128×8×28×28	256×8×28×28	256×128×3×3×3
conv3b	256×8×28×28	256×8×28×28	256×256×3×3×3
conv4a	256×4×14×14	512×4×14×14	512×256×3×3×3
conv4b	512×4×14×14	512×4×14×14	512×512×3×3×3
conv5a	512×2×7×7	512×2×7×7	512×512×3×3×3
conv5b	512×2×7×7	512×2×7×7	512×512×3×3×3
fc6	8192	4096	8192×4096
fc7	4096	4096	4096×4096
fc8	4098	$n_{output\_dim} = 10$	4096×10
Total		$n_{output\_neuron} = 23390218$	$n_{weight} = 78025792$
Total number of devices	78025792×2=156051584		

IV: Supplementary References

1 Shan, W. *et al.* A 510-nW Wake-Up Keyword-Spotting Chip Using Serial-FFT-Based MFCC and Binarized Depthwise Separable CNN in 28-nm CMOS. *IEEE Journal of Solid-State Circuits* **56**, 151-164 (2021). https://doi.org:10.1109/JSSC.2020.3029097

2 Giraldo, J. S. P., Lauwereins, S., Badami, K. & Verhelst, M. Vocell: A 65-nm Speech-Triggered Wake-Up SoC for 10-μW Keyword Spotting and Speaker Verification. *IEEE Journal of Solid-State circuits* **55**, 868-878 (2020).

3 Li, Q. *et al.* MSP-MFCC: Energy-Efficient MFCC Feature Extraction Method With Mixed-Signal Processing Architecture for Wearable Speech Recognition Applications. *IEEE Access* **8**, 48720-48730 (2020). https://doi.org:10.1109/ACCESS.2020.2979799

4 Jo, J., Yoo, H. & Park, I.-C. Energy-efficient floating-point MFCC extraction architecture for speech recognition systems. *IEEE Transactions on Very Large Scale Integration Systems* **24**, 754-758 (2016).

5 Ramos-Lara, R., López-García, M., Cantó-Navarro, E. & Puente-Rodriguez, L. Real-time speaker verification system implemented on reconfigurable hardware. *Journal of Signal Processing Systems* **71**, 89-103 (2013).

6 Haofeng, K., Weijia, S., Lane, I. & Chong, J. Efficient MFCC feature extraction on Graphics Processing Units. In Constantinides International Workshop on Signal Processing. 1-4 (IET, 2013).

7 Techpowerup. *GPU Database of NVIDIA GeForce GTX 580*, <https://www.techpowerup.com/gpu-specs/geforce-gtx-580.c270> (2010).

8 Manikandan, J., Venkataramani, B., Girish, K., Karthic, H. & Siddharth, V. Hardware Implementation of Real-Time Speech Recognition System Using TMS320C6713 DSP. In 24th Internatioal Conference on VLSI Design. 250-255 (IEEE, 2011).

9 TexasInstruments. *TMS320C6711D, C6712D, C6713B Power Consumption Summary*, <https://www.ti.com/lit/pdf/spra889 > (2005).

10 Intel. *The Specification of Intel® Pentium® 4 Processor 1.50 GHz*, <https://www.intel.com/content/www/us/en/products/sku/27423/intel-pentium-4-processor-1-50-ghz-256k-cache-400-mhz-fsb/specifications.html> (2002).

11 Buschmann, F. *et al. Pattern-Oriented Software Architecture, Volume 1, A System of Patterns*. (John Wiley & Sons, 1996).

12 Lee, J., Park, J., Kim, K. L. & Nam, J. Sample-level deep convolutional neural networks for music auto-tagging using raw waveforms. In Proceedings of the 14th Sound and Music Computing Conference (SMC). 220-226 (Michigan Publishing, 2017).

13 Lai, S., Xu, L., Liu, K. & Zhao, J. Recurrent convolutional neural networks for text classification. In Proceedings of the AAAI conference on artificial intelligence. 2267-2273 (AAAI Press, 2015).

14 Vaswani, A. *et al.* Attention is all you need. In 31st Conference on Neural Information Processing Systems. 1-11 (Association of Computational Machinery, 2017).

15 Lecun, Y., Bottou, L., Bengio, Y. & Haffner, P. Gradient-based learning applied to document recognition. *Proceedings of the IEEE* **86**, 2278-2324 (1998). https://doi.org:10.1109/5.726791

16 Tran, D., Bourdev, L., Fergus, R., Torresani, L. & Paluri, M. Learning spatiotemporal features with 3D convolutional networks. In Proceedings of the IEEE International Conference on Computer Vision. 4489-4497 (IEEE, 2015).

17 Wu, Z. *et al.* 3D ShapeNets: A deep representation for volumetric shapes. In Proceedings of the IEEE Conference on Computer Vision and Pattern Recognition. 1912-1920 (IEEE, 2015).

18 Maturana, D. & Scherer, S. Voxnet: A 3D convolutional neural network for real-time object recognition. In 2015 IEEE/RSJ International Conference on Intelligent Robots and Systems (IROS). 922-928 (IEEE, 2015).

19 Zhao, M. *et al.* Characterizing Endurance Degradation of Incremental Switching in Analog RRAM for Neuromorphic Systems. In 2018 IEEE International Electron Devices Meeting (IEDM). 20.22.21-20.22.24 (IEEE, 2018).

20 Kim, S. *et al.* Experimental Demonstration of a Second-Order Memristor and Its Ability to Biorealistically Implement Synaptic Plasticity. *Nano Letters* **15**, 2203-2211 (2015). https://doi.org:10.1021/acs.nanolett.5b00697

21 Kim, S., Choi, S. & Lu, W. Comprehensive Physical Model of Dynamic Resistive Switching in an Oxide Memristor. *ACS Nano* **8**, 2369-2376 (2014). https://doi.org:10.1021/nn405827t

22 Leighton, P. A. Electronic Processes in Ionic Crystals (Mott, N. F.; Gurney, R. W.). *Journal of Chemical Education* **18**, 249 (1941). https://doi.org:10.1021/ed018p249.1

23 Panzer, M. A. *et al.* Thermal Properties of Ultrathin Hafnium Oxide Gate Dielectric Films. *IEEE Electron Device Letters* **30**, 1269-1271 (2009). https://doi.org:10.1109/led.2009.2032937

24 Landon, C. D. *et al.* Thermal transport in tantalum oxide films for memristive applications. *Applied Physics Letters* **107**, 023108 (2015). https://doi.org:10.1063/1.4926921

25 Capron, N., Broqvist, P. & Pasquarello, A. Migration of oxygen vacancy in HfO_2 and across the HfO_2 / SiO_2 interface: A first-principles investigation. *Applied Physics Letters* **91**, 192905 (2007). https://doi.org:10.1063/1.2807282

26 Larentis, S., Nardi, F., Balatti, S., Gilmer, D. C. & Ielmini, D. Resistive Switching by Voltage-Driven Ion Migration in Bipolar RRAM—Part II: Modeling. *IEEE Transactions on Electron Devices* **59**, 2468-2475 (2012). https://doi.org:10.1109/ted.2012.2202320

27 Pahinkar;, D. G. *et al.* Computational Investigation of Nanoscale Memristor Devices for Neuromorphic Computing. In 2019 18th IEEE Intersociety Conference on Thermal and Thermomechanical Phenomena in Electronic Systems (ITherm). 318-319 (IEEE, 2019).

28 Lavasani, H. M., Pan, W., Harrington, B., Abdolvand, R. & Ayazi, F. A 76dBΩ 1.7GHz 0.18µm CMOS tunable transimpedance amplifier using broadband current pre-amplifier for high frequency lateral micromechanical oscillators. In 2010 IEEE International Solid-State Circuits Conference - (ISSCC). 318-319 (IEEE, 2010).

29 Brooks, L. & Lee, H.-S. A Zero-Crossing-Based 8-bit 200 MS/s Pipelined ADC. *IEEE Journal of Solid-State Circuits* **42**, 2677-2687 (2007). https://doi.org:10.1109/jssc.2007.908770

30 Idros, N., Rosli, A., Abdul Aziz, Z. A., Rajendran, J. & Marzuki, A. A 1.8 V high-speed 8-bit hybrid DAC with integrated rail-to-rail buffer amplifier in CMOS 180 nm. *Microelectronics International* **38**, 46-54 (2021). https://doi.org:10.1108/MI-10-2020-0073
